# Chloroplastic protein PORC undergoes heat-induced condensation and enhances thermotolerance in Arabidopsis

**DOI:** 10.1093/plphys/kiag220

**Published:** 2026-04-16

**Authors:** Fatema Alquraish, Israel Maruri-López, Marcin Luzarowski, Itzell E Hernández-Sánchez, Monika Chodasiewicz

**Affiliations:** Biological and Environmental Science and Engineering Division (BESE), King Abdullah University of Science and Technology (KAUST), Thuwal 47000, Saudi Arabia; Biological and Environmental Science and Engineering Division (BESE), King Abdullah University of Science and Technology (KAUST), Thuwal 47000, Saudi Arabia; Core Facility for Mass Spectrometry and Proteomics, Center for Molecular Biology of Heidelberg University (ZMBH), DKFZ-ZMBH Alliance, Heidelberg 69120, Germany; Biological and Environmental Science and Engineering Division (BESE), King Abdullah University of Science and Technology (KAUST), Thuwal 47000, Saudi Arabia; Biological and Environmental Science and Engineering Division (BESE), King Abdullah University of Science and Technology (KAUST), Thuwal 47000, Saudi Arabia

## Abstract

Chloroplast stress granules (cpSGs) are emerging as dynamic suborganellar condensates that play a crucial role in mediating stress response in plants. In this study, we demonstrate that the chlorophyll biosynthesis enzyme protochlorophyllide oxidoreductase C (PORC) localizes to cpSGs in response to acute and prolonged heat stress in Arabidopsis (*Arabidopsis thaliana*). While PORC promoter activity was developmentally regulated and remained unresponsive to heat, PORC protein re-localized from a diffuse chloroplast distribution into punctate structures under elevated temperatures. This condensation was reversible, translation-dependent, and absent under optimal growth conditions. Genetic disruption of PORC resulted in compromised thermotolerance, whereas overexpression enhanced photosynthetic recovery following both acute (42 °C) and prolonged (35 °C) heat stress. High-throughput phenotyping and chlorophyll fluorescence imaging confirmed enhanced Photosystem II (PSII) efficiency and increased post-stress growth rate. Proteomic profiling of heat-induced PORC-cpSGs revealed functional enrichment of photosystem I/II components, proteases (eg, FtsH), and proteins involved in chlorophyll biosynthesis and photoprotection, suggesting a stress-protective role of cpSG under heat. These findings establish PORC as a key player in the chloroplast stress response, implicating cpSGs as protective hubs that facilitate the maintenance of photosynthetic integrity under elevated temperatures. Our study provides insight into chloroplast-specific biomolecular condensates, enhancing our understanding of plant stress resilience and paving the way for future studies on the regulation of their dynamics. Additionally, it highlights how components such as PORC could be utilized to develop heat-tolerant crops.

## Teaser

The chlorophyll biosynthesis enzyme protochlorophyllide oxidoreductase C is a key player in the chloroplast stress response.

## Introduction

Biomolecular condensates have emerged as fundamental components of cellular organization, participating in diverse molecular pathways and stress responses (Emenecker et al. 2020). These dynamic, membrane-less condensates are not restricted to a specific organelle, and they have been observed across various cellular compartments, including the chloroplast (Chodasiewicz et al. 2020; Ouyang et al. 2020; Zheng et al. 2021; Londoño Vélez et al. 2022). In this organelle, biomolecular condensates have been implicated in optimizing photosynthetic efficiency and regulating protein transport. For example, condensation of ribulose-1,5-bisphosphate carboxylase/oxygenase (Rubisco) within the pyrenoid matrix of green algae enhances photosynthetic efficiency and biomass accumulation by facilitating CO_2_ fixation (Barrett et al. 2021). In land plants, ankyrin repeat proteins such as STT1 and STT2 form chloroplast-localized condensates (STT bodies) involved in cargo transport from the envelope to the thylakoid membrane (Ouyang et al. 2020; Zheng et al. 2021).

Stress granules (SGs) represent a prominent class of membrane-less condensates composed of proteins, messenger RNAs, and small molecules (Kosmacz et al. 2019; Maruri-López et al. 2021). While traditionally associated with the cytoplasm, SGs have also been observed in the chloroplast. Early evidence for plastidial RNA granules emerged in green algae under oxidative stress (Uniacke and Zerges (2008), and more recently, chloroplast SGs (cpSGs) were identified in *Arabidopsis thaliana* during acute heat stress Chodasiewicz et al. (2020). Proteomic profiling of *A. thaliana* cpSG revealed the presence of 88 proteins including several key enzymes involved in chlorophyll biosynthesis, suggesting a potential role for these condensates in regulating important plastidial processes during stress adaptation.

One of the proteins identified in cpSGs was protochlorophyllide oxidoreductase C (PORC), a nuclear-encoded enzyme essential for chlorophyll biosynthesis (Chodasiewicz et al. 2020). In Arabidopsis, the POR family consists of 3 structurally related, yet differentially regulated proteins: PORA, PORB, and PORC. These isoforms catalyze the light-dependent reduction of protochlorophyllide to chlorophyllide, a critical and culminating step in the chlorophyll biosynthetic pathway. (Oosawa et al. 2000; Su et al. 2001; Frick et al. 2003; Masuda et al. 2003; Zhang et al. 2019). The regulation of the chlorophyll biosynthesis process is complex and tightly regulated by different factors, including genetic regulation, biochemical and environmental factors, such as light availability/intensity, low or high temperature, or changes in water availability (Liu et al. 2017; Yu et al. 2024).

Although POR proteins have been characterized for their roles in chlorophyll biosynthesis and light signaling (Oosawa et al. 2000; Masuda et al. 2003; Masuda and Takamiya 2004), their potential involvement in stress adaptation has only recently begun to emerge. The physiological significance of PORC localization to cpSGs, its impact on stress resilience, and the broader functional landscape of cpSGs remain poorly understood. We hypothesize that PORC recruitment to cpSG may be linked to the enhanced capacity of plants to withstand heat stress. To confirm this hypothesis, we combined genetic, physiological, and proteomic approaches. We demonstrated that PORC relocalizes into heat-induced cpSGs. We further showed that PORC is required for efficient photosynthetic recovery and survival following acute and prolonged heat stress. Moreover, proteomic analysis of purified PORC-containing cpSGs revealed the selective enrichment of photosynthesis-related proteins, chlorophyll biosynthesis enzymes, and proteolytic machinery, supporting a functional role for cpSGs in safeguarding chloroplast homeostasis during stress adaptation. Our findings uncover a putative way by which a core metabolic enzyme contributes to the heat stress response through biomolecular condensation, revealing an unexplored layer of regulation within the chloroplast.

## Results

### High temperature induces PORC localization into cpSGs

**Figure 1:**
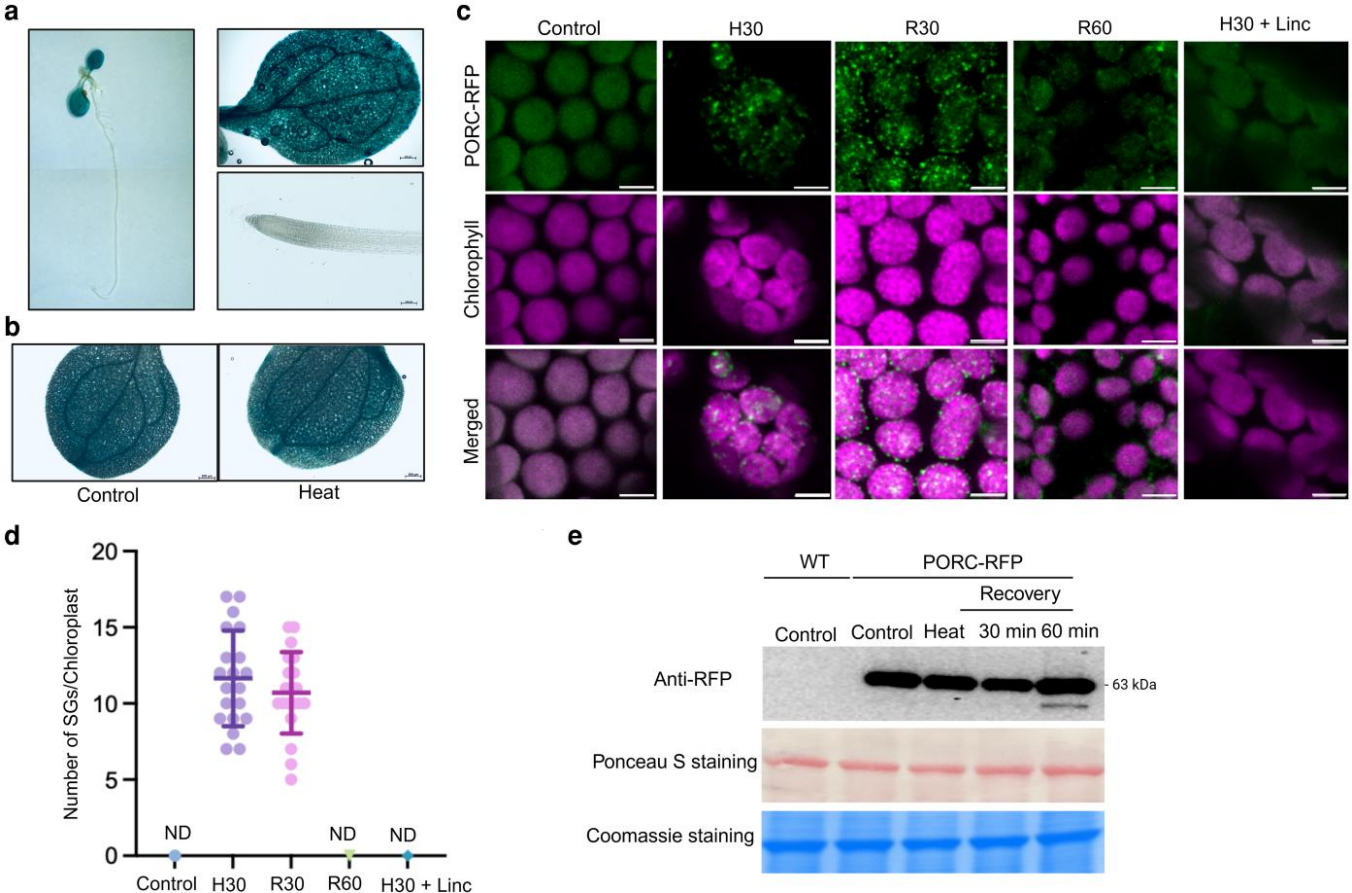
PORC localizes into SGs under high temperature stress. a) PORC promoter activity visualized by GUS staining of 7-d-old *A. thaliana* plants presented in a composite image of the whole seedlings on the left panel. b) PORC promoter activity under control and heat stress conditions (42 °C for 30 min) visualized by GUS staining. c) PORC-RFP localization under control and heat stress conditions (42 °C) in stable *A. thaliana* transgenic lines and under 2 time points of the recovery phase, 30 and 60 min. Linc—Lincomycin. Scale bar = 5 µm. d) Quantification of SGs per chloroplast. n = 20 data presented as mean and standard deviation. (ND: nondetermined, H30: heat 30 min, R30: recovery 30 min, R60: recovery 60 min, Linc—Lincomycin). e) Western blot analysis of PORC-RFP level at different time points using anti-RFP antibody. Coomassie-stained SDS–PAGE gel is shown as a loading control.

To evaluate *PORC* gene expression patterns, we generated Arabidopsis transgenic lines with the PORC promoter fused to the β-glucuronidase (GUS) reporter gene. In 7-d-old seedlings under non-stress conditions, the promoter-driven GUS expression showed tissue-specific signal in the shoot apical meristem, hypocotyl, cotyledons, and leaves, with no detectable signal in the root (Fig. 1a). We further examined whether differential expression patterns could be observed upon heat stress. To investigate this, we subjected 7-d-old seedlings to 42 °C for 30 min. Our results indicate that PORC is expressed specifically in leaves and its expression remained unchanged between the 2 conditions (Fig. 1b). This suggests that PORC expression is mainly controlled by the developmental stage rather than by short-term heat stress (Fig. S1) (Klepikova et al. 2016).

To investigate the protein dynamics of the PORC protein in vivo, the stable transgenic *A. thaliana* lines expressing the PORC-RFP construct were generated. Seven-d-old seedlings were subjected to either control or heat stress conditions and subsequently analyzed by confocal microscopy. PORC-RFP showed diffused fluorescent signals in the chloroplast under control conditions. However, upon exposure to heat (42 °C for 30 min), PORC formed punctate-like structures in the chloroplast (Fig. 1c). In contrast, RFP expressed alone remained diffusely distributed in the cytosol upon heat stress (Fig. S2a), supporting the specificity of the PORC signal. Since it has been shown that mRNA is essential for cpSG formation, a property shared with previously characterized cSGs and cpSGs (Chodasiewicz et al. 2020; Maruri-López et al. 2021), to further confirm the nature of observed condensates, we evaluated the mechanism of cpSG formation by using lincomycin. Lincomycin is an inhibitor of chloroplast translation that binds the 50S ribosomal subunit, preventing peptide bond formation, thereby inhibiting mRNA sequestration into cpSGs (Chotewutmontri and Barkan 2018). Lincomycin treatment inhibited the condensation property of PORC protein under heat treatment (Fig. 1c), suggesting that the presence of translationally repressed mRNA is required for PORC-cpSGs formation.

Cytosolic SGs are known to be dynamic, rapidly forming upon exposure to stress, and disassembling after the stress relief (Maruri-López et al. 2021; Allen and Strader 2022). To investigate whether chloroplast-localized PORC condensates follow a similar pattern, we evaluated the PORC dynamics under the recovery phase following heat stress. To achieve this, plants were first exposed to 42 °C for 30 min and then transferred to room temperature to allow for the recovery from the stress. During the first 30 min of recovery, the number of PORC-RFP puncta decreased, and by 60 min, the granules had fully disassembled (Fig. 1c and 1d). To determine whether the disappearance of PORC-containing cpSGs during recovery was due to granule disassembly rather than a decrease in protein abundance, PORC-RFP protein levels were assessed by western blot analysis before, during the heat stress, and throughout the recovery period, using an anti-RFP antibody. Our results showed that protein levels remained consistent throughout the tested timeframe, confirming that the condensates indeed disassemble and PORC diffuses from the condensates (Fig. 1e).

To evaluate the stress specificity of PORC-containing cpSGs, the formation of PORC-cpSG was evaluated in response to other abiotic stresses. Salinity and drought stress are 2 of the most dominant abiotic stresses that can negatively affect plant growth and development (Dos Santos et al. 2022). To mimic these conditions, seedlings were treated with 0.5 M NaCl (salinity stress) and 0.5 M sorbitol (osmotic stress to simulate drought). In addition, considering that abscisic acid (ABA) is a central regulator of abiotic stress signaling and functionally linked to chlorophyll biosynthesis, we also tested whether ABA treatment could induce cpSG formation. Our observations showed that all 3 conditions tested triggered a noticeable change in PORC-RFP protein behavior, as it began to localize toward the edges of the chloroplasts. However, these stress-induced structures did not resemble the canonical morphology of cpSGs observed under heat stress (Fig. S2b), suggesting a heat stress-specific response.

As previously mentioned, PORC belongs to the family of 3. The POR genes (PORA, B, and C) encode proteins with over 75% amino acid sequence identity. Phylogenetic analysis indicates that these proteins diverged following a duplication event, with PORA and PORB sharing a closer evolutionary relationship with each other than with PORC (Fig. S3a). To assess whether PORA and PORB form punctate structures, similar to those observed for PORC, we transiently expressed PORA-RFP and PORB-RFP in *N. benthamiana* leaves. Under control conditions, both protein fusions exhibited a diffuse signal. However, upon heat treatment, both PORA and PORB began to form punctate structures resembling the morphology of cpSGs (Fig. S3b and S3c).

### Genetic disruption and overexpression of *PORC* affect the thermotolerance and photosynthetic recovery of *A. thaliana* plants

Given the involvement of POR proteins in chlorophyll biosynthesis and their heat-induced relocalization into cpSGs, we examined the PORC contribution to plant thermotolerance using loss- and gain-of-function lines. Due to the fact that *PORB* and *PORC* exhibit similar global expression patterns, which may account for the functional redundancy previously reported in the literature (Frick et al. 2003; Masuda et al. 2003; Klepikova et al. 2016), we generated mutations in *PORC* and *PORB* through targeted gene editing. In the *porc* single mutant, a biallelic heterozygous mutation was introduced, consisting of a nucleotide deletion in one allele and a substitution in the other. In the *porb/porc* double mutant lines, PORC carried a biallelic homozygous deletion, while *PORB* exhibited a biallelic heterozygous deletion (Fig. S4a). All mutations resulted in frameshifts and premature stop codons, and are predicted to produce truncated, non-functional protein products (Fig. S4a). Successful disruption of *PORC* and *PORB* genes was confirmed by PCR and sequencing in their respective mutant backgrounds. In addition, RT-qPCR analysis revealed a significant reduction in *PORC* and *PORB* transcript levels in the respective mutant backgrounds compared to Col-0 wild-type (WT) (Fig. S4b and S4c).

To assess the phenotypic effects of *PORC* and *PORB* disruption, the generated mutant backgrounds were evaluated under normal growth conditions and compared to WT and a PORC overexpression line (PORC-OE). As a first approach, germination rate was examined, revealing a significant reduction in the *porc* mutant compared to WT, PORC-OE, and *porc/porb* double mutant. After 2 d, germination of all genotypes reached its peak at >90%, except for *porc* mutant, which remained at ∼70% (Fig. S4d). Despite the role of PORC in chlorophyll biosynthesis, total chlorophyll content measured in 10-d-old plants did not differ among all tested backgrounds (Fig. S4e). Additionally, primary root length in 7-d-old plants showed no significant variation between the mutants and control lines (Fig. S4f).

**Figure 2:**
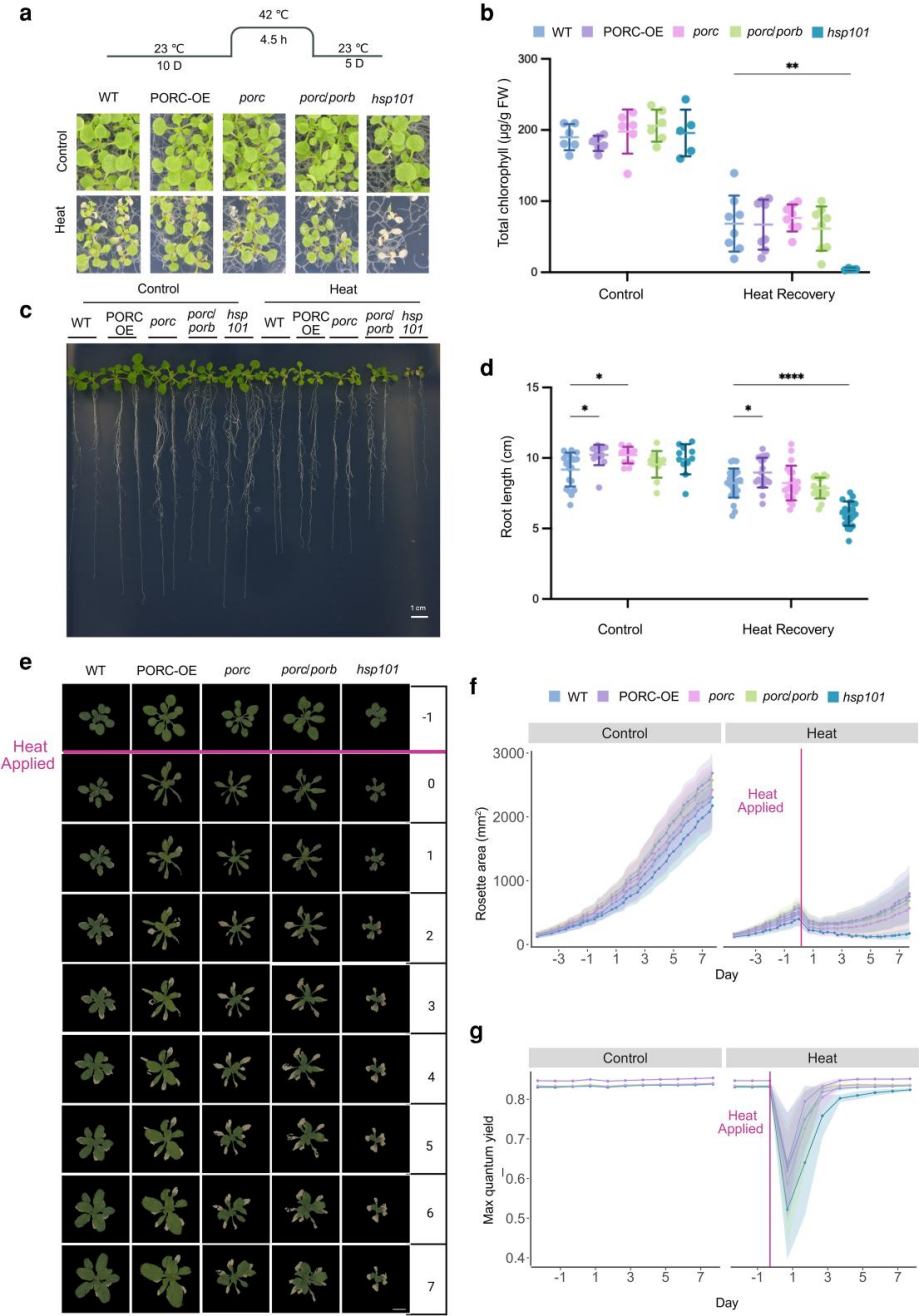
PORC is important for heat stress response in plants. a) Schematic representation of the heat stress experiment performed at the seedling stage and the phenotype of heat stress-treated plants after 5 d of recovery. D: day. b) Chlorophyll content of plants from (a). Graph represents mean values and standard deviation (SD). (n varies between 5 and 6 for individual groups with each of a population of 15 seedlings, **P ≤ 0.01 Two-way ANOVA test). FW: fresh weight, WT: Col-0 wild-type. c) Phenotype analysis of root development of Arabidopsis WT, and *porc* transgenic lines after heat shock treatment. Graph represents mean values and SD (n = 25, *P ≤ 0.05, ****P < 0.0001, Two-way ANOVA test). d) Quantifications of the root length. Graph represents mean values and SD (n = 25, *P ≤ 0.05, ****P < 0.0001, Two-way ANOVA test). e) Schematic representation of heat stress experiment setup for adult *A. thaliana* plants. D: Day and time series of RGB images of representative plants before the heat stress (−1), after (0) and during the 7-d recovery period of heat-treated plants. Images were digitally extracted for comparison. f) Graph represents measurement of the Rosette area presented as mean and SD for plants subjected to heat stress conditions *vs* control plants (n = 18). g) Graph represents a comparison of maximum quantum yield (QY_max_) of control and 8 h heat-stressed plants (n = 18 plants/treatment).

Since there were no significant differences among all tested backgrounds under control conditions, we aimed to determine if challenging plants with heat stress would reveal differences in their performance. First, the heat shock treatment was performed by exposing 10-d-old plants of the WT, *porc, porc/porb*, PORC-OE, and heat shock protein 101 (*hsp101*) backgrounds to 42 °C for 5 h, followed by 5 d of recovery at 23 °C (Fig. 2a). After 5 d of stress recovery, leaf bleaching was observed across all genotypes; particularly, in the *hsp101* mutant, 100% of seedlings failed to recover and died (Fig. 2a), indicating the effectiveness of our treatment. Interestingly, new leaf emergence and chlorophyll re-accumulation were observed in WT, *porc*, *porb/porc*, and PORC-OE. However, the total chlorophyll content did not differ significantly among those genotypes (Fig. 2b). Approximately 85% of WT, *porc*, *porb/porc*, PORC-OE seedlings resumed normal growth, while the remaining ∼15% exhibited mild to severe damage or failed to recover (Fig. 2b). Chlorophyll levels are closely associated with root length and overall plant development (Van Gelderen et al. 2018). To investigate the significance of PORC in root growth and its response to heat stress, we analyzed root length after heat shock recovery. Consistent with chlorophyll content, heat stress led to a reduction in root growth, which was more pronounced in the *hsp101* mutant (Fig. 2c and 2d, Fig. S5a and S5b). PORC overexpression lines exhibited significantly longer roots under both control and heat recovery conditions, suggesting better plant performance. In addition to physiological parameters, we focused on determining stress-related parameters. It is well known that under heat stress, plants accumulate reactive oxygen species (ROS), which not only cause oxidative stress but also serve as crucial signaling molecules that activate protective pathways against environmental challenges (Medina et al. 2021; Hendrix et al. 2023). Thus, we investigated whether manipulation of PORC abundance can affect the scavenging of ROS produced under heat stress. We achieved this by measuring hydrogen peroxide levels using 3,3′-diaminobenzidine (DAB) staining. Exposure to heat shock resulted in ROS accumulation, as evidenced by comparable levels of DAB staining in both 7- and 10-d-old seedlings. (Fig. S5c and S5d).

To assess vegetative-stage phenotypes in adult plants, we employed high-throughput phenotyping, a non-invasive approach for monitoring plant morphology and photosynthetic performance. Three-wk-old plants of WT, *porc* mutant*, porc/porb* double mutant, PORC-OE, and *hsp101* genotypes were grown in the Photon Systems Instruments (PSI) growth room and subjected to heat stress treatment of 45 °C for 8 h in a preheated Percival growth chamber. Next, plants were moved back to recover in the PSI growth room for 7 d. Different parameters were employed to evaluate plant fitness, including chlorophyll fluorescence, red-green-blue (RGB), and thermal imaging. RGB images were obtained to track changes in traits reflecting plant growth (Fig. 2e, Fig. S6a). With consideration for the circadian rhythms, measurements were taken 3 times per day, beginning 1 d prior to heat treatment and continuing throughout the 7-d recovery period.

All genotypes exhibited growth inhibition reflected as a reduction of rosette size after heat stress. Among them, *porc* and *porc/porb* mutants displayed smaller rosette size (∼500 mm^2^) in comparison to WT and PORC-OE (∼900 mm^2^); with *hsp101* (∼200 mm^2^) showing the most severe reduction (Fig. 2f). Plant photosynthetic performance was evaluated using the light curve protocol described previously by Gao et al. (2020) with stepwise light exposure of cool-white actinic light with increasing intensity of 95, 210, 320, 440, 555, and 670 μmol m^−2^ s^−1^ (Fig. S6b). The efficiency of Photosystem II (PSII) was calculated as the maximum quantum yield (QY_max_). We observed a sharp decrease in QY_max_ immediately following the heat stress treatment, which was fully recovered by 3 to 4 d post-treatment. Interestingly, the PORC-OE transgenic lines outperformed all other lines, showing a notable increase under control and heat conditions where QY_max_ of photosynthesis was more efficient (Fig. 2g). This was further supported by the electron transport rate (ETR), where PORC-OE exhibited the highest rates among all tested genotypes (Fig. S6c). Together, these results indicate that PORC plays a key role in maintaining plant fitness under heat stress conditions.

### Overexpression of PORC improves tolerance to prolonged heat stress conditions

**Figure 3:**
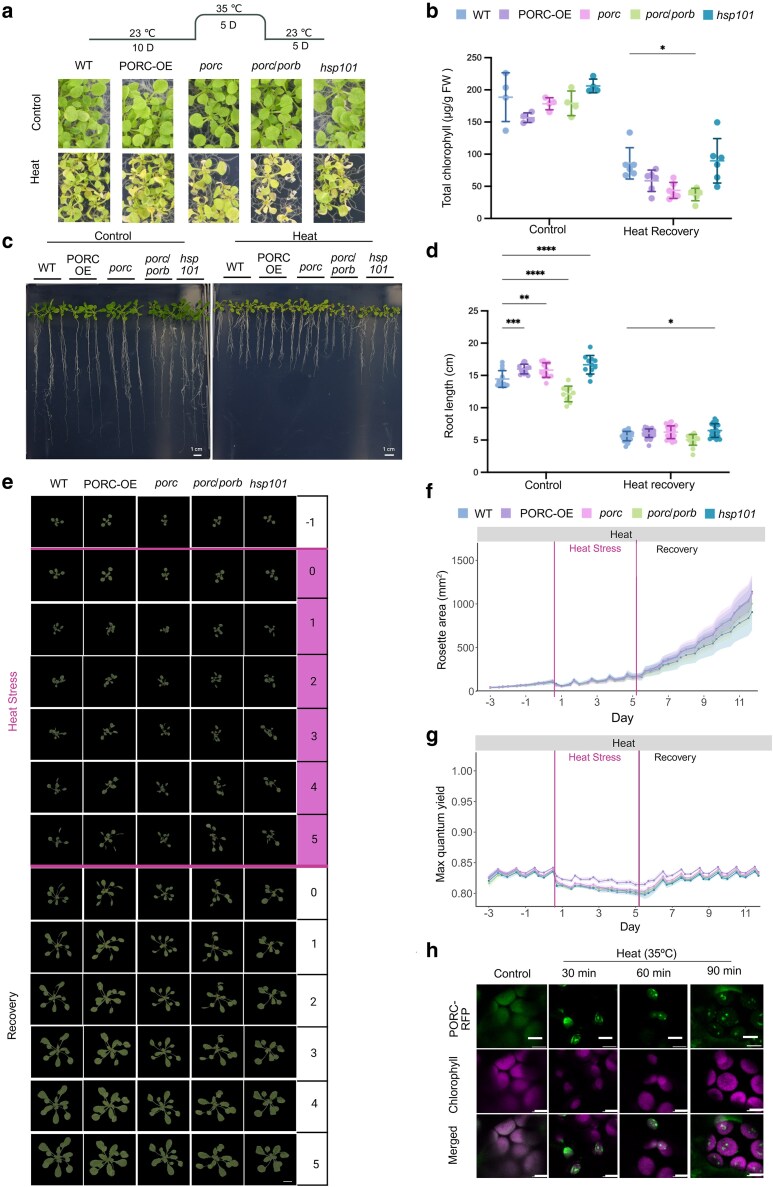
PORC is important for survival of prolonged heat stress conditions. a) Experimental setup for prolonged heat treatment at seedling stage. D: Day and phenotype of plants treated with prolonged heat stress. Photos are taken after 5 d of recovery. b) Chlorophyll content measured in plants from (a). Graph represents mean values and SD (n varies between 4 and 8 for individual groups with each of a population of 15 seedlings, *: *P* ≤ 0.05, Two-way ANOVA followed by Tukey's post hoc test). FW: fresh weight, WT: Col-0 wild-type. c) Phenotype analysis of root development of Arabidopsis wild type, and *porc* transgenic lines after heat shock treatment. d) Quantifications of the root length. Graph represents mean values and SD. (n = 25, *P ≤ 0.05, ****P < 0.0001, Two-way ANOVA test). e) High-throughput time-course imaging of *PORC-OE, porc, porc/porb*, *hsp101*, and Col-0 plants (n = 26) under control, prolonged stress, and recovery phase. Images were digitally extracted for comparison. f) Graphs represent mean and SD for Rosetta size expressed as area, under control, prolonged heat, and recovery conditions (n = 26). g) Maximum quantum yield (QY_max_) of PORC lines before, during, and after heat stress, including the recovery phase (n = 26). h) Cellular dynamics of PORC under 35 °C (heat stress) and recovery conditions. n = 5. Scale bar = 5 µm.

Given that our results suggested that PORC contributes to plant recovery after acute heat stress, we next evaluated whether it also plays a role in tolerance to prolonged high-temperature exposure. Prolonged heat is known to impair photosynthesis by damaging chloroplast structures and reducing photosynthetic efficiency (Peng et al. 2024). To assess the role of PORC under prolonged heat stress, 10-d-old plants of WT, *porc, porb/porc*, PORC-OE, and *hsp101* genotypes were subjected to extended heat treatment at 35 °C for 5 d, followed by a 5-d recovery period under normal growth conditions (Fig. 3a). Chlorophyll measurements revealed reduced content in all genotypes after heat stress, with a significant decline observed in *porb/porc* mutants (Fig. 3b). These results suggest that disruption of PORC, particularly in combination with PORB, compromises chlorophyll stability under sustained heat stress. We further analyzed the root length following the heat recovery. In line with our previous findings under heat shock stress and with the chlorophyll content results, prolonged heat stress led to growth arrest reflected as the roots' length reduction in comparison to the control condition by 40% (Fig. 3c and d, Fig. S7a and S7b). To gain insight into whether plant fitness after heat stress is directly influenced by ROS accumulation, we measured hydrogen peroxide levels. Under control conditions, PORC-OE lines accumulated less ROS than the WT (Fig. S7c and S7d). After prolonged heat exposure, ROS levels were reduced relative to the control, with greater accumulation observed in *porc/porb* mutants. These results indicate that the loss of *PORC/PORB* impairs the ability of plants to scavenge ROS during extended heat stress.

To investigate the effects of prolonged heat stress at the vegetative stage, we analyzed adult plants using similar imaging and physiological parameters as described for the heat shock experiments, with modifications to accommodate extended heat exposure. Nine-day-old seedlings were grown in the PSI phenotyping platform and, at 15 d old, plants were subjected to prolonged heat treatment at 35 °C for 5 d, followed by a 5-d recovery period (Fig. 3e). Consistent with our earlier findings under acute stress, PORC-OE lines exhibited larger rosette size (Fig. 3f, Fig. S8a) and significantly higher QY_max_ and electron transport rate values compared to all other genotypes during the heat stress and early stages of recovery (Fig. 3g, Fig. S8b).

We previously demonstrated that PORC relocalizes to cpSGs in response to acute heat stress; however, it remained unclear whether similar condensate formation occurs under milder, prolonged conditions. Given the enhanced thermotolerance of PORC-OE lines during prolonged stress, we hypothesized that cpSG formation might also occur at 35 °C. To test this, 7-d-old seedlings expressing PORC-RFP were exposed to 35 °C for increasing duration of time. Results revealed that PORC-containing granules gradually formed under these conditions, becoming clearly visible after 90 min of heat exposure (Fig. 3h).

### Proteomic profiling of purified cpSG reveals enrichment of photosynthesis- and stress-associated proteins

To determine the protein composition of PORC-containing cpSGs, we isolated cpSG-enriched and soluble fractions from 35S:PORC-RFP *A. thaliana* seedlings subjected to acute heat stress (42 °C for 30 min). Fractionation was performed by differential centrifugation (Xie et al. 2023) followed by immunoprecipitation using anti-RFP magnetic Sepharose beads (Kosmacz and Skirycz 2020). As a control for nonspecific protein interactions, we also included heat-treated plants expressing RFP alone (Fig. S9a). All fractions were subjected to mass spectrometry (MS)-based proteomic analysis.

**Figure 4:**
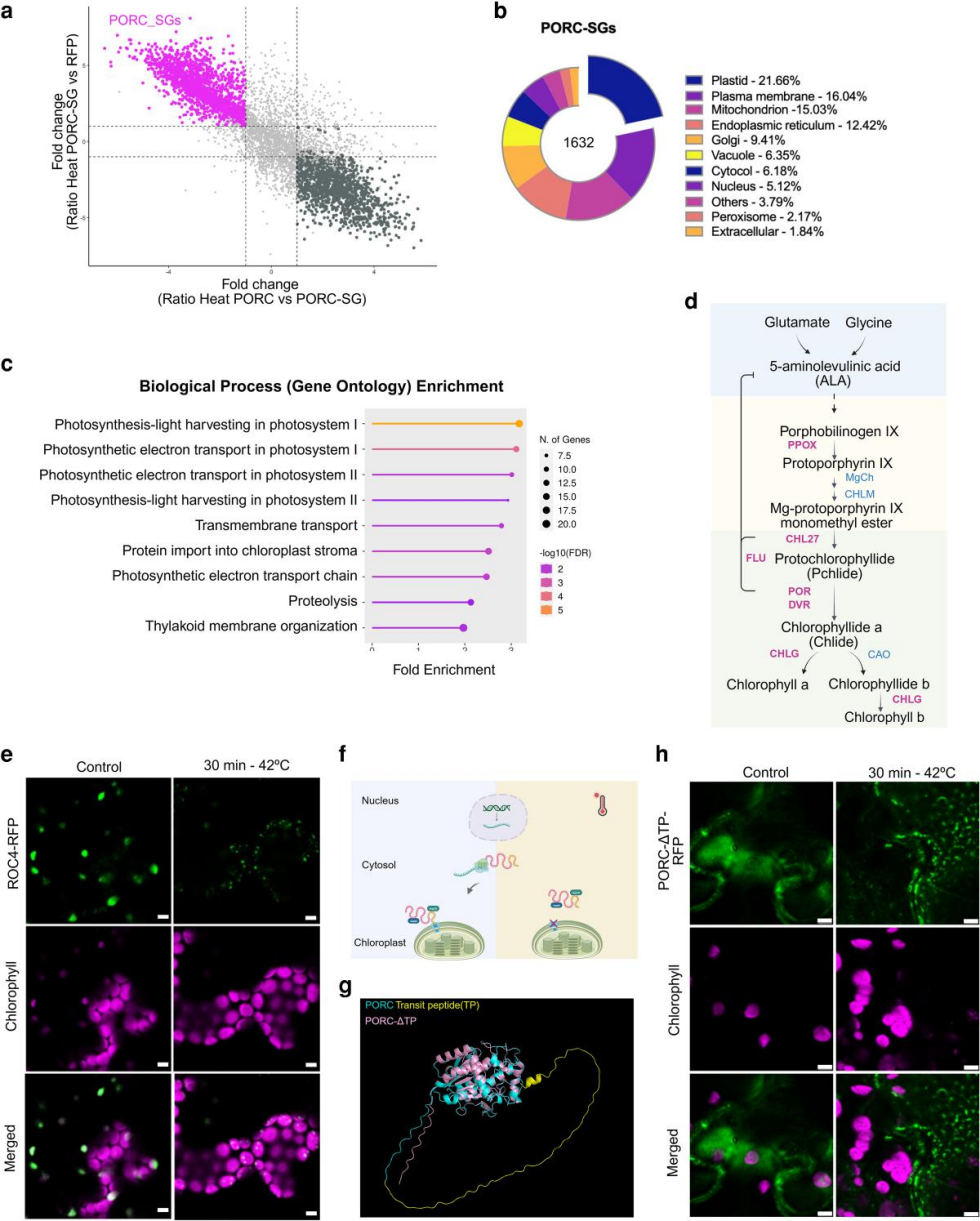
PORC condensates are rich in proteins involved in photosynthesis. a) Scatter plot representing proteins enriched in PORC cpSGs. PORC-SG: PORC-RFP insoluble fraction (cpSG), PORC: PORC-RFP soluble fraction, RFP: RFP-only control line soluble fraction. Fold change (log_2_): the ratio of protein abundance between the indicated conditions. b) A summary of the predicted subcellular localization of proteins found in PORC-cpSGs. c) Biological process enrichment of plastidial proteins found in PORC cpSG. d) A simplified schematic representation of chlorophyll biosynthesis, highlighting proteins enriched in the cpSG isolate in magenta, while blue indicates other enzymes involved in the process but not enriched. Abbreviations of the enzymes: PPOX: protoporphyrinogen oxidase, CHL27: Magnesium-protoporphyrin IX monomethyl ester [oxidative] cyclase. FLU: fluorescent in blue light, POR: protochlorophyllide oxidoreductase. DVR: divinyl chlorophyllide an 8-vinyl-reductase. CHLG: chlorophyll synthase. MgCH; magnesium chelatase, CHLM: Magnesium protoporphyrin IX methyltransferase, CAO: chlorophyllide an oxygenase. e) Subcellular localization of ROC4-RFP protein in stable *A. thaliana* lines. Scale bar = 5 µm. f) Heat impact on nuclear encoded chloroplast proteins transport: Heat stress disrupts the transport of nuclear-encoded chloroplast proteins, leading to impaired translocation and retention of these proteins within the cytosol. g) PyMOL alignment of AlphaFold predication of PORC WT vs PORC-ΔTP. h) Confocal images of PORC-ΔTP dynamics in tobacco leaves. Scale bar = 5 µm.

To identify cpSG-enriched proteins, PORC-cpSG interactors were obtained by comparing the PORC soluble and cpSG-enriched fractions and refined by excluding general RFP interactors for specificity. Proteomics analysis resulted in the identification of 1,632 putatively cpSG-associated proteins (Fig. S9b and S9c), which were further analyzed for subcellular localization using the SUBA database (Hooper et al. 2017) (Fig. 4a). Here, 389 proteins corresponding to the biggest group of proteins of 21.66% were affiliated as localized to the plastid. Interestingly, the remaining proteins were distributed across different cellular compartments, including the plasma membrane (16.04%), mitochondrion (15.03%), endoplasmic reticulum (12.42%), and cytosol (6.11%), among others (Fig. 4b). Since the primary objective of this experiment was to identify PORC interactors within cpSGs, we focused solely on plastidial proteins. Among the 389 proteins identified, 363 are nuclear, while 26 are chloroplast-encoded. The GO term enrichment analysis (Ge et al. 2020) revealed photosynthesis-light harvesting in photosystem I (PSI) and photosystem II (PSII) with FDR = 1.4E−06 and 5.0E−02, respectively, in addition to proteolysis with FDR = 4.5E−02 (Fig. 4c) as the most enriched pathways. These pathways included core proteins from the light-harvesting complex (LHC), proteins involved in photoprotection and proteolysis, and proteins preventing cell death under high-intensity light conditions. As an example, 14 different proteases were identified, 8 of which belong to the filamentation temperature-sensitive protein H (FtsH) protease family (Dataset S1). FtsH proteases play a crucial role in the repair and degradation of damaged D1 protein in PSII, contributing to the maintenance of thylakoid membrane integrity and overall photosynthetic function (Lu 2016). Moreover, proteins involved in the chlorophyll biosynthetic process were identified (Fig. 4d). The presence of multiple light-harvesting proteins, FtsH proteases, and biosynthetic enzymes suggests that cpSGs may serve as protective reservoirs to stabilize key chloroplast functions during acute heat stress and facilitate rapid recovery upon stress relief.

One of the proteins detected in cpSG under acute temperature stress was rotamase CYP 4 (ROC4), a unique cyclophilin, present in the chloroplast stroma in *A. thaliana* (Lippuner et al. 1994). ROC4 protein has been shown to play an important role in catalyzing correct folding and integration of proteins during the repair process in chloroplasts, particularly, D1 subunit from PSII (Cai et al. 2008). Elevated temperature stress is challenging for photosystem activity; therefore, it is considered to be very detrimental for photosynthesis. To validate whether ROC4 can localize in cpSG upon heat stress, *A. thaliana* lines expressing 35S:ROC4-RFP were generated. In detail, leaves of 7-d-old seedlings exposed to control and high temperature conditions (42 °C for 30 min) were visualized under confocal microscopy. ROC4-RFP was homogeneously localized in the chloroplast under control while under heat stress conditions, its localization changed to puncta structures resembling the morphology of cpSGs (Fig. 4e). These results suggest that ROC4 is a condensate and possibly recruited to cpSGs in response to acute heat stress, potentially as a protective mechanism to preserve its role in PSII repair and facilitate rapid reintegration upon stress recovery.

### Heat induces PORC aggregation in the cytosol

Our proteomic analysis identified 111 proteins enriched in PORC-cpSG that exhibit cytosol localization, in addition to proteins that localized to other compartments, where their presence in the chloroplast appears implausible due to the absence of known transport mechanisms. Interestingly, plastid-localized proteins have been previously reported within cSGs (Kosmacz et al. 2019; Gutierrez-Beltran et al. 2021). The majority of plastidial proteins are synthesized in the nucleus, followed by translation in the cytoplasm and subsequent transport to the chloroplast with the assistance of a transient peptide (TP). Heat stress has been shown to disrupt this transport, potentially leading to failed protein translocation (Dutta et al. 2009; Wang et al. 2023) (Fig. 4f). We hypothesize that this disruption may impair chloroplast protein import, allowing chloroplast-targeted proteins to transiently accumulate in the cytosol.

To further investigate the underlying cause of this recurring phenomenon, we sought to modify PORC localization by mimicking the situation under heat and generating a scenario where PORC protein cannot enter the chloroplast. Thus, we decided to generate a PORC version lacking the TP (PORC-ΔTP), which is responsible for chloroplast targeting. Using AlphaFold (Jumper et al. 2021), we predicted the structure of the PORC-ΔTP, and using PyMOL(The PyMOL Molecular Graphics System, Version 1.2r3pre, Schrödinger, LLC), we aligned it with wild-type PORC (Fig. 4g). The deletion of the TP did not alter the overall 3D conformation of the protein but significantly reduced the length of the low-complexity sequence, which is attributed to the TP presence. To evaluate its behavior in vivo, we cloned the PORC-ΔTP version into the pGWB554 vector to transiently express it in tobacco leaves. As expected, under normal conditions, PORC-ΔTP exhibited a diffuse cytoplasmic signal, failing to localize to chloroplasts (Fig. 4h). Notably, upon heat treatment, PORC-ΔTP began to aggregate, suggesting PORC formed puncta structures independently of its subcellular targeting localization and enabled shared interactions with both cSGs and cpSGs.

## Discussion

The cpSGs are emerging as crucial components of plastidial stress responses, yet their composition, dynamics, and functional roles are still unclear (Allen and Strader 2022; Gutierrez-Beltran et al. 2015; Gutierrez-Beltran et al. 2021; Hamada et al., 2018; Kearly et al. 2022; Kosmacz et al. 2019; Kosmacz and Skirycz 2020; Maruri-López et al. 2021; Xie et al. 2023). This study highlights a role for the chlorophyll biosynthesis enzyme PORC in cpSG formation and thermotolerance. Here, we showcase that *PORC* is not transcriptionally upregulated by heat stress, indicating that its contribution to stress response is likely regulated at the post-translational level or through condensate formation. We demonstrate that PORC relocalizes into dynamic, translation-dependent condensates in response to heat stress, and that these structures disassemble upon recovery without degradation of PORC protein. This suggests a reversible stress adaptation mechanism, consistent with previous research by Ha and colleagues (2017), which demonstrated that POR protein levels are stably maintained at elevated temperatures during the autotrophic development stage.

Elevated temperatures can negatively impact photosynthesis in plants by disturbing key processes within the photosynthetic machinery. PSII is considered one of the major stress-vulnerable sites in the photosynthetic machinery that is prone to damage (Allakhverdiev et al. 2008). To mitigate thermal stress, plants must develop adaptive mechanisms that protect their photosynthetic systems. Several studies showed that after heat stress, photosynthesis efficiency relies heavily on the protein biosynthetic machinery to repair thermal or photodamage of PSII (Allakhverdiev et al. 2008; Sakata et al. 2013; Hu et al. 2020). Following this line, using high-throughput phenotyping, we demonstrated that PORC-OE line exhibited multiple functional traits associated with improved adaptability to heat stress (Guo et al. 2022; Gu, 2023; Scafaro et al. 2023; Neri et al. 2024). The enhanced performance during heat stress and early stages of recovery can be attributed to their ability to maintain higher QY_max_ in adult plants. This is further supported by elevated electron transport rates, indicating that PSII is functioning more efficiently, enabling sustained energy conversion and reduced photodamage under heat stress. In contrast, *porc/porb* double mutants displayed reduced performance and a lower chlorophyll content at the seedling stage. Notably, both PORC and PORB appear to play a role in the thermotolerance protective mechanism, as the phenotype was only observed in the double mutant and not in the single *porc* mutant, further supporting their functional redundancy in heat stress adaptation.

In line with our previous results, the proteomic profiling of purified PORC-cpSGs uncovered a significant enrichment for photosynthesis and light-harvesting processes in PSI and PSII, as reflected in the Gene Ontology (GO) analysis. Notably, the presence of all major LHC proteins from both PSI (LHCAs) and PSII (LHCBs) was identified. Additionally, proteases, such as FtsH1 and FtsH2. (Lindahl et al. 2000; Sakamoto et al. 2003; Zhang et al. 2024), which are essential for the degradation of damaged D1 protein in the photosystem II were identified. We hypothesize that PORC-containing cpSGs play a protective role in safeguarding essential photosynthetic proteins for recovery. Heat stress can compromise membrane integrity, leading to the dissociation of LHC from the membranes and its subsequent recruitment into PORC-containing cpSGs due to its crucial role in recovery. Once the stress is alleviated, cpSGs disassemble, releasing proteins that can be efficiently reintegrated when needed. This suggests that cpSGs contribute to protein stability and facilitate enhanced recovery under stress conditions by rapidly mobilizing proteins, thereby conserving both time and energy.

In chloroplasts, proteases are typically dispersed across specific compartments, primarily associated with membranes (Nishimura et al. 2016). In our study, we identified 14 different proteases enriched in cpSGs. Heat stress can impact membrane fluidity and integrity by altering lipid composition, potentially leading to membrane leakage. A study by Bussi et al., (2023) in mammalian cells demonstrated that cytosolic SGs are recruited to damaged membranes, acting as molecular plugs to stabilize and facilitate repair. It is plausible that a similar mechanism operates in chloroplasts, which could be an explanation for the presence of proteases in cpSGs.

These findings raise the possibility that cpSGs may act as specialized biochemical microenvironments that regulate enzymatic efficiency under stress. Biomolecular condensates create a protective environment that facilitates enzymatic activity by concentrating enzymes and substrates, thereby enhancing catalytic efficiency under specific conditions. Cells not only benefit from the increased enzyme concentration within condensates, which can reach up to 1,000-fold, but the crowded environment also influences catalytic dynamics and substrate affinity (O’Flynn and Mittag 2021). The enrichment of multiple enzymes involved in the chlorophyll biosynthesis pathway within cpSG and their presence in proximity suggests that cpSG perhaps acts as a reaction hub to concentrate enzymes and substrates, optimizing biochemical reactions under heat stress.

Among the proteins identified in heat-induced PORC-cpSGs, ROC4 was identified as a notable component due to its dual chaperone and reductase functions, which are critical for protein refolding and stress response regulation in plants (Liu et al. 2020). Mutants lacking ROC4 display hypersensitivity to oxidative stress caused by high salt, osmotic shock, and prolonged exposure to high light (Dominguez-Solis et al. 2008; Speiser et al. 2012; Liu et al. 2020). Cai et al., (2008) demonstrated that ROC4 is essential for facilitating the proper folding and integration of damaged D proteins during PSII repair, highlighting its significant role during the reparation process in chloroplasts. In this study, we confirm that ROC4 relocalizes to cpSGs under heat stress conditions.

PORC, like many other chloroplast-localized proteins, is nuclear-encoded. More than 3,000 chloroplast proteins are synthesized in the cytosol and contain an N-terminal transit peptide that facilitates their translocation into the chloroplast (Bruce 2000; Nellaepalli et al. 2023; Xing et al. 2025). This translocation is mediated by the TOC–TIC system, where proteins first pass through the Translocon at the Outer (TOC) chloroplast membrane complex, then are transferred to the Translocon at the Inner (TIC) chloroplast membrane complex, and finally undergo further processing inside the chloroplast (Bruce 2000; Nellaepalli et al. 2023; Xing et al. 2025). However, heat stress can disrupt the TOC–TIC system, leading to impaired protein transport. Numerous studies have shown that heat stress downregulates the expression of genes involved in the TOC–TIC system (Dutta et al. 2009; Wang et al. 2023). Additionally, heat affects membrane integrity, causing instability in the import apparatus, which leads to a decrease in import efficiency (Dutta et al. 2009). As a result, chloroplast-targeted proteins may be unable to translocate properly, leading to their transient accumulation in the cytosol. We propose that this impairment may explain the cytosolic interactors identified in our SG isolate, supported by the observation that PORC-ΔTP constructs cytosolic aggregates under heat stress. Together, these findings suggest that mislocalized chloroplast proteins may associate with cytosolic stress cSGs when import into the chloroplast is compromised. Whether this interaction serves functions beyond cellular protection remains to be determined.

**Figure 5:**
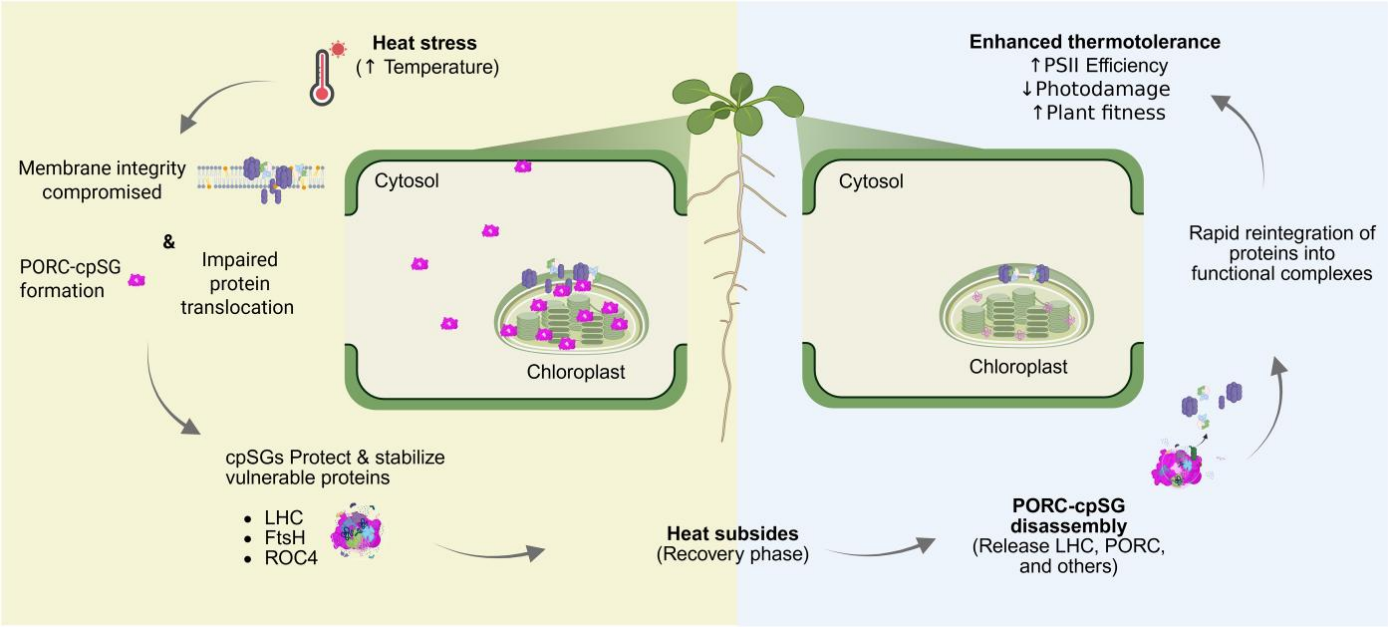
Proposed model of how PORC contributes to plant heat stress response. Left panel. Heat stress disrupts membrane integrity, impairs protein translocation, and triggers cpSG formation. cpSGs act as protective hubs that stabilize vulnerable chloroplast proteins. Right panel. When heat stress subsides, these proteins can be rapidly reintegrated into their functional complexes, enhancing thermotolerance. This recovery process improves PSII efficiency, reduces photodamage, and ultimately increases overall plant fitness.

In conclusion, our study identified Arabidopsis PORC as a dynamic component of heat-induced cpSGs that form during heat stress and disassemble upon recovery. Genetic and phenotypic analyses indicated that PORC knockout mutants are hypersensitive to heat stress, whereas PORC-overexpressing lines display enhanced heat tolerance. Proteomic profiling of PORC-containing cpSGs revealed an enrichment of proteins involved in photosynthesis, chlorophyll biosynthesis, and proteolysis. These combinations suggest that cpSG assembly helps protect the photosynthetic machinery, buffering chloroplasts against stress-induced damage and supporting protein quality control mechanisms, facilitating the repair of damaged and misfolded proteins during heat stress. Together, our findings support a functional link between cpSG dynamics and plant thermotolerance and suggest that PORC contributes to the coordination of chloroplast protein homeostasis during heat stress. These results indicate that reversible, stress-induced chloroplast granules may represent an adaptive mechanism to preserve chloroplast function under adverse conditions (Fig. 5). POR plays an essential role in chlorophyll biosynthesis, a function that is evolutionarily conserved among organisms originating from cyanobacteria. In angiosperms, however, the number and identity of POR isoforms vary between species, including important cereal crops such as wheat and barley (Masuda and Takamiya 2004; Gabruk and Mysliwa-Kurdziel 2015; Vedalankar and Tripathy 2024). More broadly, this work deepens our understanding of chloroplast stress responses and provides a foundation for future studies investigating how biomolecular condensate dynamics are regulated and whether specific components, such as PORC, can be harnessed to improve heat tolerance in plants.

## Materials and methods

### Plant material and growth conditions

Seeds of *A. thaliana* Col-0 ecotype were used as control line for this study. PORC promoter::GUS, PORC-RFP, ROC4-RFP, and RFP tag alone seeds were surface sterilized with a 20% bleach solution for 5 min while shaking, followed by 5 min in 70% ethanol containing Tween 20. After sterilization, the seeds were thoroughly washed 5 times with sterile Milli-Q H_2_O before sowing. Seeds were grown in square Petri dishes on 0.5× Murashige and Skoog (MS) growth medium, pH 5.7, containing 1% (w/v) sucrose and 1% (w/v) agar (Murashige and Skoog 1962). Seeds were stratified for 2 to 3 d at 4 °C in the dark, and then they were incubated in a vertical position at 22 ± 2 °C in a growth chamber with a 16-h photoperiod (120 μmol m^−2^ s^−1^).

### Generation of constructs for promoter activity and subcellular localization

To evaluate the tissue-specific expression of *PORC* gene (At1G03630), a 910 bp fragment upstream of the start codon was synthetically generated as the native promoter sequence. This promoter was flanked with BsaI restriction sites and appropriate overhangs for cloning into the GreenGate entry vector pGGA000 (Lampropoulos et al. 2013) via *Bsa*I digestion and T4 DNA ligase-mediated ligation. The final expression construct was assembled into the pGGZ001 destination vector using the GreenGate system, by combining the following modules, PORC promoter (pGGA000), a default 5′ UTR sequence (pGGB003), the GUS reporter gene (pGGC051), a linker with stop codon (pGGD002), the UBQ10 terminator (pGGE009), and a Hygromycin resistance cassette (pGGF005).

For subcellular localization studies, the full-length *PORC* and *ROC4* (AT3G62030) coding sequence were PCR-amplified using Taq Phusion High-Fidelity (Thermo Fisher Scientific, USA) and directionally cloned into the pENTR/D-TOPO (Thermo Fisher Scientific, USA). The subcloning procedure was carried out by site-specific recombination using Gateway LR Clonase II Enzyme Mix (Invitrogen, USA) according to the manufacturer's instructions. For stable transformation of *A. thaliana*, the PORC and ROC4 entry clones were recombined into the pGWB554 vector. The *PORA* (AT5G54190) and *PORB* (AT4G27440) coding sequences were synthetically generated with flanking attL1 and attL2 recombination sites for subsequent Gateway cloning into the pGWB554 vector. All the generated constructs were verified by DNA sequencing. To express the RFP tag alone as a control, the Gateway-compatible vector pH7RWG2 was used.

The resulting expression constructs were introduced into *Agrobacterium tumefaciens* strain GV3101 by electroporation. For the PORC promoter::GUS construct, GV3101 cells complemented with the pSOUP plasmid were used to support T-DNA replication from the pGreen-based vector.

### Transient expression in *N. benthamiana* leaves

To visualize POR protein localization, transient expression assays were performed in *N. benthamiana*. *Agrobacterium tumefaciens* GV3101 strains carrying the constructs pGWB554-PORC, pGWB554-PORA, pGWB554-PORB, and pGWB554-PORC-ΔTP were cultured overnight at 28 °C in LB medium supplemented with appropriate antibiotics. The cultures were centrifuged at 4,000 *×g* for 15 min, and the bacterial pellets were resuspended in infiltration buffer containing 10 mM MES (pH 5.6), 10 mM MgCl_2_, and 0.2 mM acetosyringone. The suspensions were incubated for 3 h in a rocking platform at room temperature. Fully expanded leaves of 6-wk-old *N. benthamiana* plants were infiltrated using a needleless syringe. Plants were kept in the dark for 2 to 3 d post-infiltration before imaging for subcellular localization.

### Stable transformation of *A. thaliana*

Stable transformation of *A. thaliana* was performed using the floral dip method as described by Zhang et al. (2006), with minor modifications. Cells of *A. tumefaciens* GV3101 strains carrying the binary constructs (PORC promoter::GUS, PORC-RFP, ROC4-RFP, and RFP tag alone) were grown in LB medium containing appropriate antibiotics and harvested by centrifugation at 4,000 *×g* for 15 min. Bacterial pellets were resuspended in infiltration medium consisting of 0.5× MS, pH 5.7, 5% (w/v) sucrose, and 0.02% (v/v) Silwet L-77. Flowering *A. thaliana* plants were dipped into the suspension for 30 s with gentle agitation, then covered and kept in high humidity overnight. Transformed seeds (T1 generation) were harvested and selected on 0.5× MS, pH 5.7, containing 1% (w/v) sucrose and 1% (w/v) agar supplemented with 50 µg/mL hygromycin. Homozygous T3 lines were identified and used for all subsequent experiments.

### Generation of PORC and PORB CRISPR-Cas9 mutant lines

Targeted mutagenesis of *A. thaliana* PORC and PORB was performed using the CRISPR/Cas9 system. For each gene, 3 single-guide RNAs (sgRNAs) were designed to target early coding regions to induce frameshift mutations. The U6-gRNAs expression cassettes were assembled into the pBE12.1-zmpl-tRNA(K21) vector by Gibson assembly (Gibson et al. 2009). Constructs were transformed into *A. tumefaciens* strain GV3101 and introduced into *A. thaliana* Col-0 plants via the floral dip method (Zhang et al. 2006). T1 transformants were selected on 0.5× MS Basta-containing medium. Gene editing events were identified in T2 and T3 generations by PCR amplification and Sanger sequencing of the target regions.

### GUS histochemical analysis for promoter activity

To evaluate the activity of the PORC promoter under control and heat stress conditions, 7-d-old transgenic *A. thaliana* seedlings expressing the PORC promoter::GUS construct were subjected to histochemical staining. GUS staining was performed using x-Gluc (Thermo Fisher Scientific, USA). Briefly, seedlings were incubated for 12 h at 37 °C in staining buffer containing 0.7 mg/mL 5-bromo-4-chloro-3-indolyl-β-D-glucuronide (X-Gluc) in 200 mM sodium phosphate buffer (pH 7.0), 100 mM potassium ferricyanide, and 100 mM potassium ferrocyanide. Following staining, tissues were cleared by incubation in 70% ethanol at room temperature with gentle shaking for 6 h. Promoter activity was assessed in seedlings grown under control conditions and in seedlings exposed to heat stress (42 °C for 30 min) immediately prior to staining. At least 15 seedlings per condition were analyzed using a Zeiss Axioskop 2 microscope at 10× magnification. Each experiment was independently repeated at least 3 times with consistent results.

### Confocal imaging

Transgenic *A. thaliana* lines were imaged using a high-resolution inverted laser scanning confocal microscope (Leica SP8, Leica Microsystems, Germany). Images were acquired using a 63 × oil immersion objective at 512 × 512-pixel resolution with a line averaging of 6. RFP fluorescence was excited at 561 nm and detected between 595 and 620 nm. Chlorophyll autofluorescence was recorded using 670 nm excitation and a detection range of 675 to 719 nm. The laser power was set to 7%, with a detector gain of 375.5 and a pinhole diameter of 1.5 Airy unit (AU). The image analysis and cpSGs quantifications were done using Fiji (ImageJ) software (Schindelin et al. 2012).

### Germination assay

Germination rate was evaluated in *A. thaliana* Col-0 wild type, *porc* mutant, *porc/porb* double mutant, and PORC-OE lines under control growth conditions, as described above. Seeds were scored every 24 h for 4 consecutive days, and germination was defined as visible radicle emergence. Each genotype was analyzed using at least 3 biological replicates (18 = seeds per replicate).

### Chlorophyll extraction and quantification

Total chlorophyll was extracted from the shoot tissue of *A. thaliana* seedlings of different genotypes under control and heat stress conditions. Fresh weight was recorded prior to extraction, and tissue was incubated overnight in 80% (v/v) ethanol at room temperature in darkness. Total chlorophyll content in the extract was quantified spectrophotometrically using a multimode plate reader (Varioskan Flash, Thermo Scientific, USA) by measuring absorbance at 664 and 647 nm. Total chlorophyll concentration (µg/g fresh weight) was calculated using the formula: Total chlorophyll = [7.93(A_664_) + 19.53(A_647_)]/fresh weight (Hiscox and Israelstam 1979).

### Primary root length assay

Primary root length was recorded in *A. thaliana* seedlings of different genotypes grown under control conditions. Seedlings were grown vertically, and root growth was allowed for 7 d after germination. Plates were scanned on Day 7, and primary root length was measured using Fiji (ImageJ) software (Schindelin et al. 2012). Each genotype was analyzed with at least 3 biological replicates (n = 10 seedlings per replicate).

### Hydrogen peroxide detection assay

3,3′-diaminobenzidine (DAB) (Thermo Fisher Scientific, USA). was used to determine ROS accumulation. Ten plants per genotype were analyzed under each condition. Staining was performed as previously described. Plants were incubated in 1 mg/mL DAB solution in the dark with gentle shaking for 4 h, after which the solution was replaced with a bleaching solution (ethanol: acetic acid: glycerol, 3:1:1) for 12 h prior to visualization.

### Heat treatments

Heat stress experiments were conducted under both acute and prolonged conditions, depending on the assay. For subcellular localization analysis, 7-d-old *A. thaliana* seedlings expressing PORC-RFP were incubated at 42 °C for 30 min in preheated liquid 0.5× MS medium and then visualized by confocal microscopy. For recovery time-lapse imaging, seedlings were transferred to solid 0.5× MS medium after heat treatment, and confocal images were captured at 30- and 60-min post-stress to monitor PORC-RFP dynamics. In extended heat exposure assays, seedlings were treated at 35 °C for varying durations and imaged at 30-min intervals until cpSGs formation was observed.

For chlorophyll quantification, 10-d-old seedlings were subjected to heat shock at 42 °C for 4.5 h in the dark. For phenotypic analysis under prolonged heat stress, seedlings were exposed to 35 °C for 5 d under a 16-h photoperiod, followed by a 5-d recovery period under control conditions prior to tissue collection.

### High-throughput phenotyping

High-throughput phenotyping was performed at the PSI phenotyping platform. For acute stress, 21-d-old plants were treated at 45 °C for 8 h in a preheated Percival chamber and then transferred back to the PSI growth room for recovery under standard conditions. For prolonged stress, 15-d-old plants were exposed to 35 °C for 5 d, followed by 5 d of recovery. In both cases, measurements (eg, rosette area and photosynthetic efficiency) were taken 3 times per day, starting 3 d prior to heat treatment and continuing throughout the recovery period.

### RNA extraction and RT-qPCR

Total RNA was extracted using the Maxwell® RSC Plant RNA Kit (Promega Corporation, USA) following the manufacturer's instructions. To eliminate any genomic DNA contamination, the extracted RNA samples were treated with TURBO™ DNase (Invitrogen, USA) according to the standard DNase treatment protocol provided by the manufacturer. The absence of genomic DNA was verified using intron *MAF5* primers. Subsequently, cDNA was synthesized using SuperScript IV REVerse Transcriptase (Invitrogen, USA). The quality of the cDNA was assessed by RT-qPCR using *GAPDH* 5′ and *GAPDH* 3′ primers. cDNA from Col-0 was used as a control, and RT-qPCR was performed using PowerUp™ SYBR™ Green Master Mix (Applied Biosystems, USA).

### Protein extraction and western blotting

Total protein was extracted from 7-d-old *A. thaliana* plants subjected to control, heat and recovery treatments using ice-cold lysis buffer (50 mM Tris-HCl pH 7.4, 100 mM potassium acetate, 2 mM magnesium acetate, 0.5 mM Dithiothreitol (DTT), 1 mM NaF, 1 mM Na_3_VO_4_, 1 × protease inhibitor cocktail (Thermo Fisher Scientific, USA), and 1 U/µL RNasin (Promega Corporation, USA). The extracted proteins were then separated by pre-casted 4 to 12% SDS–polyacrylamide gel electrophoresis (Invitrogen NuPAGE Bis-Tris Mini Protein Gels), Coomassie blue staining, performed using the eStain L1 Protein Staining System (GenScript, USA) following manufacturer's instructions, served as a loading control. The proteins were subsequently transferred onto a 0.45 µm NC nitrocellulose membrane by wet electroblotting transfer method (30 V, 1 h 16 min), and successful transfer was confirmed using Ponceau S staining solution (Thermo Fisher Scientific, USA). The membrane was then blocked with 5% nonfat milk in tris-buffered saline supplemented with Tween 20 (TBST). Next, the membrane was incubated overnight at 4 °C with a horseradish peroxidase (HRP) conjugated Anti-RFP antibody (Abcam, UK). Protein detection was carried out using the ECL Prime Western Blotting System (Thermo Fisher Scientific, USA) and visualized using Bio-Rad ChemiDoc MP imaging system.

### The cpSG extraction

Enrichment of cpSG was performed from *A. thaliana* seedlings expressing PORC-RFP construct using a modified protocol based on previously described methods (Kosmacz and Skirycz 2020; Xie et al. 2023). Approximately 2 g of 10-d-old seedlings subjected heat stress conditions were homogenized in 2 mL of ice-cold lysis buffer (50 mM Tris-HCl pH 7.4, 100 mM potassium acetate, 2 mM magnesium acetate, 0.5 mM DTT, 1 mM NaF, 1 mM Na_3_VO_4_, 1 × protease inhibitor cocktail (Thermo Fisher Scientific, USA), and 1 U/µL RNasin (Promega Corporation, USA) using a pre-chilled mortar and pestle. The lysate was filtered through a 40 µm cell strainer (pluriSelect Life Science, USA) and centrifuged at 18,000*×g* for 20 min at 4 °C. The resulting pellet was resuspended in lysis buffer and further centrifuged at 800× g for 2 min at 4 °C to enrich the cpSG fraction. A volume of 375 µL from both the cpSG pellet and the corresponding supernatant was used for immunoprecipitation. cpSG enrichment was confirmed by confocal microscopy based on PORC-RFP signal localization.

For immunoprecipitation, 0.8 mL of Dynabeads Protein A (Invitrogen, USA) was equilibrated by rotation for 30 min at room temperature in 0.8 mL DEPC-treated phosphate-buffered saline (PBS, pH 7.4). Beads were magnetically separated and washed once in DEPC-treated PBS for 5 min, followed by three 5-min washes with lysis buffer. For each sample, 30 µL of pre-equilibrated Dynabeads was incubated with 370 µL of SG or supernatant fraction (supplemented with RNasin at 1:100 dilution) for 15 min at room temperature under gentle rotation. Beads were separated, and the supernatant was incubated with 10 µL of rabbit anti-RFP antibody (710530: Thermo Fisher Scientific, USA) for 1 h at room temperature with gentle rotation. Unbound antibody was removed by centrifugation at 14,000*×g* for 15 min at 4 °C, and the pellet was resuspended in 500 µL of lysis buffer supplemented with 5 µL RNasin. The antibody-bound lysate was incubated with 60 µL of pre-equilibrated Dynabeads for 15 min at room temperature. Beads were then washed 3 times for 5 min each in cold lysis buffer. After the final wash, the supernatant was discarded, leaving cpSGs bound to the Dynabeads for downstream analysis.

### Protein preparation and LC-MS/MS analysis

Bead-bound protein complexes were incubated in a denaturation buffer containing 6 M urea, 10 mM DTT, and 50 mM ammonium bicarbonate (pH 8) at room temperature for 30 min. Proteins were then alkylated with 10 mM iodoacetamide in 50 mM ammonium bicarbonate in the dark for 20 min. Proteins were digested with Trypsin/Lys-C mix (200 ng, Promega Corporation, USA) according to the manufacturer's instructions. The urea concentration was first reduced to 4 M using 50 mM ammonium bicarbonate, and samples were incubated at 37 °C for 3 to 4 h. The solution was then further diluted to 1 M urea and incubated overnight at 37 °C to complete digestion. Digestion was stopped by adding 0.1% formic acid. Peptides were desalted using Sep-Pak C18 columns (Waters, USA), vacuum-dried, and resuspended in 0.1% formic acid for liquid chromatography-mass spectrometry (LC-MS) analysis.

### MS analysis using timsTOF pro MS

Approximately 200 ng of peptide mixture per sample was analyzed using a timsTOF Pro 2 QTOF mass spectrometer coupled to a nanoElute liquid chromatography system (Bruker Daltonik GmbH, Germany). Peptides were injected directly into a reverse-phase C18 Aurora emitter column (75 μm i.d.×250 mm, 1.6 μm particle size, 120 Å pore size; Ion Opticks, Australia) using a one-column separation setup. Chromatographic separation was performed over an 80-min gradient with mobile phase A (0.1% formic acid in water) and mobile phase B (0.1% formic acid in acetonitrile) as follows: 2 to 25% B over 60 min, 25 to 37% B over 10 min, ramping from 37% to 95% B over 5 min, and holding at 95% B for an additional 5 min. The column temperature was maintained at 50 °C, with a constant flow rate of 250 nL/min. Eluted peptides were introduced into the mass spectrometer using a CaptiveSpray nano-electrospray ion source (Bruker Daltonik GmbH) operated at 1.5 kV.

MS was performed in positive ion mode with trapped ion mobility spectrometry (TIMS) enabled. The ion source temperature was set to 180 °C with a dry gas flow of 3 L/min. Data were acquired using the parallel accumulation serial fragmentation (PASEF) method (Mol Cell Proteomics, 2018, 17(12):2534 to 2545). Each acquisition cycle included 9 PASEF MS/MS scans with a total cycle time of 1.26 s. The ramp time was 120 ms, with a TIMS scan range of 0.64 to 1.45 Vs cm^−2^ (1/K_0_). Collision energy increased linearly from 20.0 eV at 0.60 (1/K_0_) to 59.0 eV at 1.60 Vs cm^−2^. The MS and MS/MS mass scan range was set to m/z 100 to 1700. TIMS accumulation time was 100 ms. Precursor selection criteria included a target intensity of 15,000 arbitrary units (au) and a minimum threshold of 1,500 au. Active exclusion duration was 0.4 min. Peak detection was performed using absolute intensity thresholds of 5,000 for both mass and mobilogram signals.

### MS data analysis

The obtained data were analyzed using MaxQuant software to determine protein intensity values (Cox and Mann 2008). The data were further processed for quality check and data imputation using R (R Core Team 2021) and RStudio (RStudio Team 2020). Violin plots were used to visualize the distribution of protein abundances across replicates, confirming the consistency and reproducibility of the acquired data. To further reduce data variation, protein intensities were median-normalized and log2-transformed. Only proteins reproducibly quantified in at least 3 replicates in at least one of the studied groups were processed further. Missing values were imputed using the MissForest algorithm (Stekhoven and Bühlmann 2012). Statistical significance between groups was determined using one-way ANOVA with *P*-values adjusted using the Benjamini–Hochberg method, followed by Tukey's post hoc test for pairwise comparisons. To obtain the list of cpSG-enriched proteins, 2 filtering steps as selection criteria were applied. First, PORC-cpSG enriched interactors were identified by comparing the PORC soluble fraction to the cpSG-enriched fraction. Proteins were considered enriched if the log2 ratio of PORC vs. PORC-cpSG was ≤−1, with a *P*-value <0.05 (Tukey HSD test). A negative fold change in the ratio PORC_vs_PORC-cpSG indicated enrichment of proteins in the cpSGs. Next, we refined the list by removing general RFP interactors. This was achieved by computing the log2 fold change between PORC-cpSG and the RFP control lines, retaining only proteins with a log2 fold change ≥1 and a *P*-value <0.05 (Tukey HSD test).

### Statistical analysis

Statistical analysis and graphical data exploration were performed using Prism (GraphPad Software, USA). Statistical tests and details are shown in the figure legends. Differences between groups were considered significant at *P-*value <0.05 unless stated otherwise.

### Accession numbers

Sequence data from this article can be found in the GenBank/EMBL data libraries under accession numbers AT1G03630 and AT3G62030

### Data deposition

Log in to the PRIDE website using the following details:

**Project accession:** PXD065110

**Token:** Lvn23XJArzrt

Alternatively, the reviewer can access the dataset by logging in to the PRIDE website using the following account details:

**Username:**  reviewer_pxd065110@ebi.ac.uk

**Password:** QvqlAzgvz68F

## Supplementary Material

kiag220_Supplementary_Data

## Data Availability

The proteomic data are deposited to PRIDE website as mentioned above.
