# Supplementary material for: Chloroplastic protein PORC undergoes heat-induced condensation and enhances thermotolerance in Arabidopsis
Source: Plant Physiol. 2026 Apr 16;201(1):kiag220. doi: 10.1093/plphys/kiag220 (PMC13191595; doi:10.1093/plphys/kiag220)
Supplement: kiag220_Supplementary_Data [file kiag220_supplementary_data.zip › Supplementary Data.pdf]

A

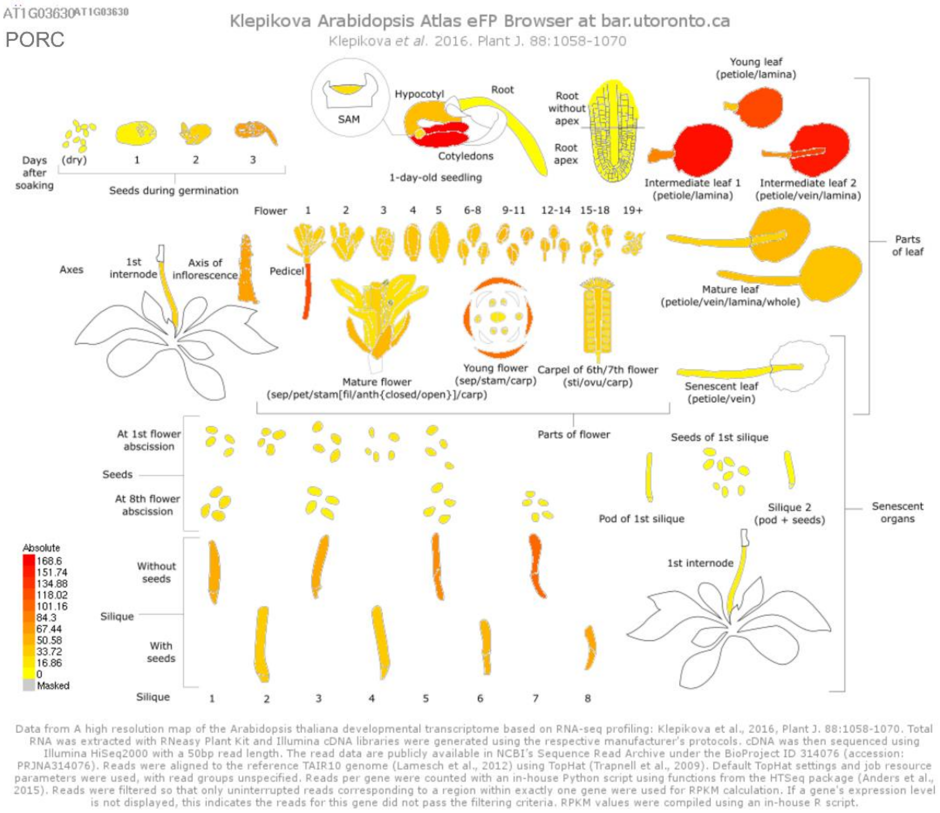

Supplementary Figure 1. Global expression pattern of *PORC* from Klepikova Atlas (Klepikova et al., 2016).

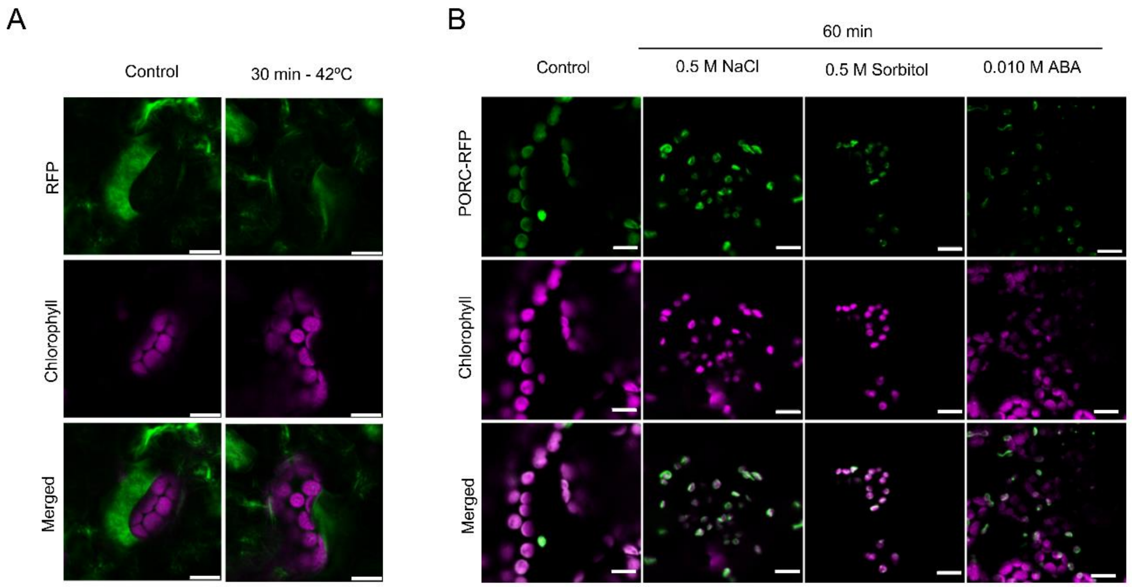

Supplementary Figure 2. Specificity of PORC-RFP reposnse. A. The RFP dynamics under heat stress. **B.** Cellular localization of PORC-RFP under different stress treatments. Seven-day old seedlings were subjected to different abiotic stresses and visualized under the confocal microscopy. Scale bar = 10 µm.

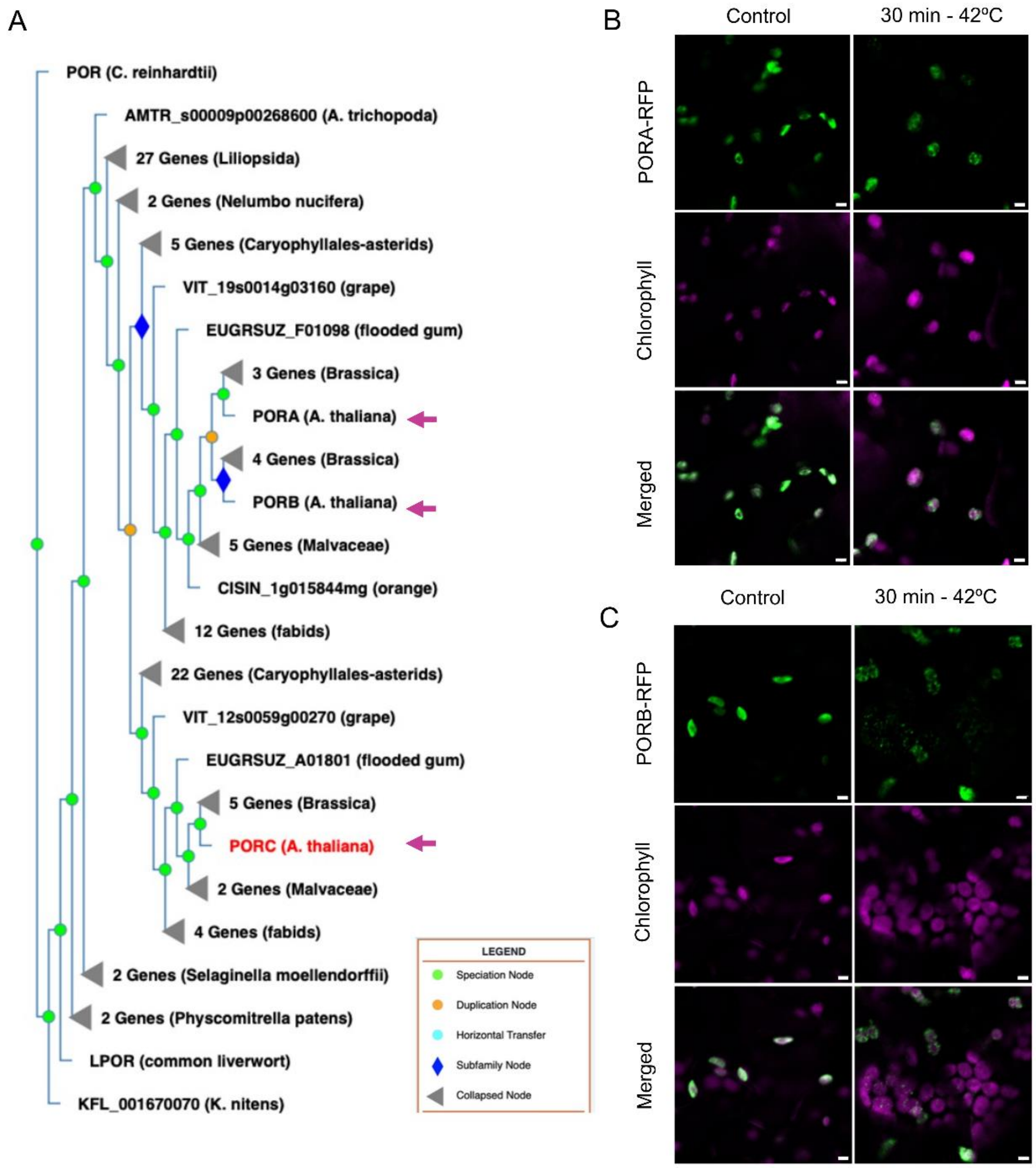

Supplementary Figure 3. Cellular localization and dynamics of PORA and PORB. A. *PORC* phylogeny tree generated by PhyloGene *(P. Zhang et al., 2020)*, *Arabidopsis* paralogs, *PORA* and *PORB* are highlighted by the pink arrow. Fluorescence microscopy images of PORA and PORB dynamics in stable Arabidopsis lines (**B**. & **C**.). Linc: lincomycin. Scale bar = 5 µm.

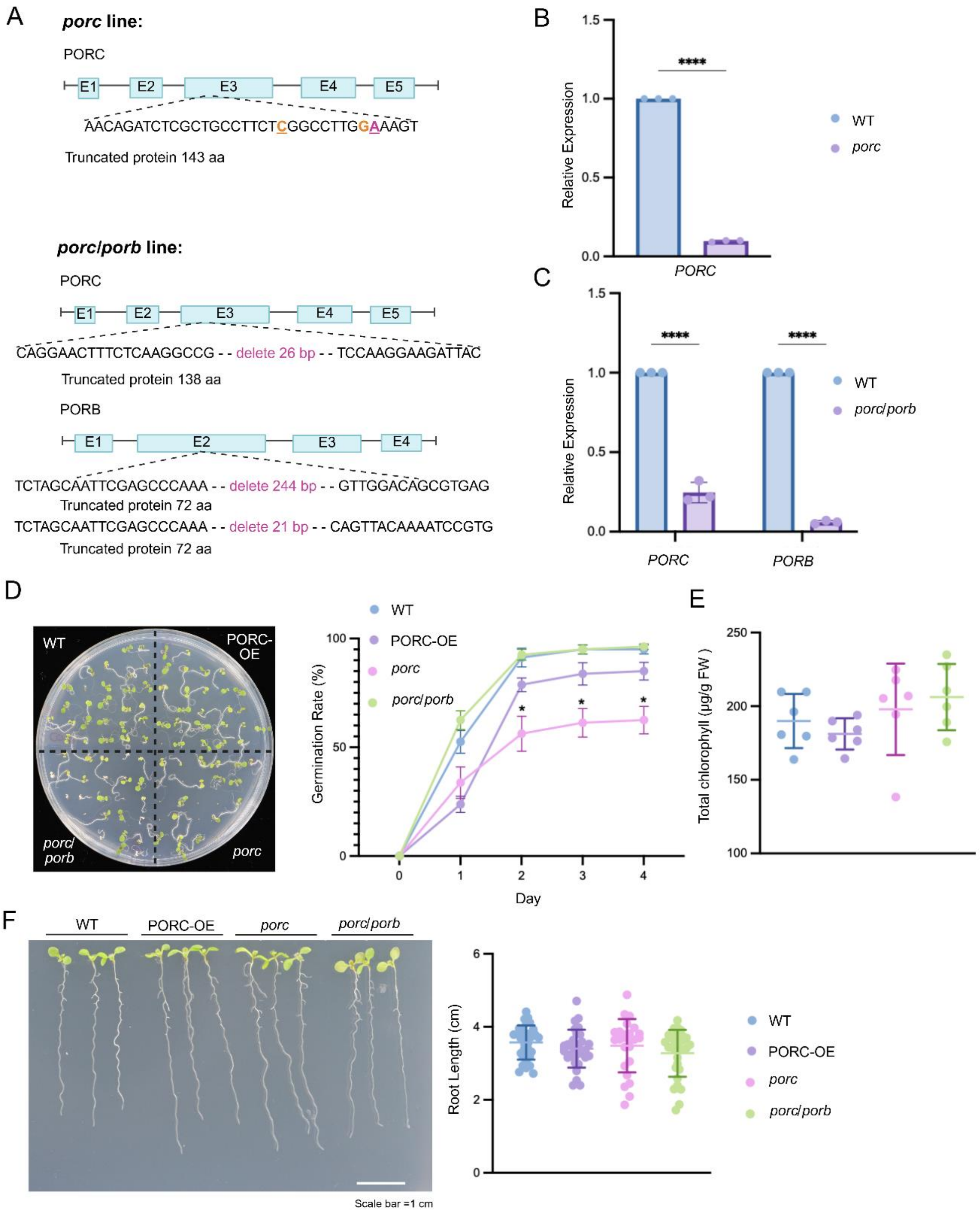

Supplementary Figure 4. Characterization of *POR* mutants' lines. **A.** Schematic representation and target sequences of *porc* and *porc/porb* mutant lines generated by CRISPR/Cas9. **B.** Relative gene expression of *PORC* single mutant lines analyzed by RTqPCR. (n=3****: *p*-value < 0.000, Two-way ANOVA) WT: Col-0 wild-type. **C**. Relative expression level of *PORB* and *PORC* in double mutant lines analyzed by RTqPCR. (n=3, ****: P value < 0.0001, Two-way ANOVA). **D.** Germination rate of POR transgenic lines observed for four days after seeds are exposed to light. Graph represents mean values and SD (ns: *p*-value ≥ 0.05, *: *p*-value= 0.01 to 0.05, Two-way

ANOVA). **E.** Chlorophyll content of fourteen-day-old seedlings. Graph represents mean values and SD (n varies between 4 and 8 for individual groups with each of a population of 15 seedlings, ns: p-value $\geq$ 0.05, One-way ANOVA) FW: fresh weight. **F.** Root length of seven-day-old seedlings. Graph represents mean values and SD. (n= 20-30 seedlings, ns: p-value $\geq$ 0.05, One-way ANOVA).

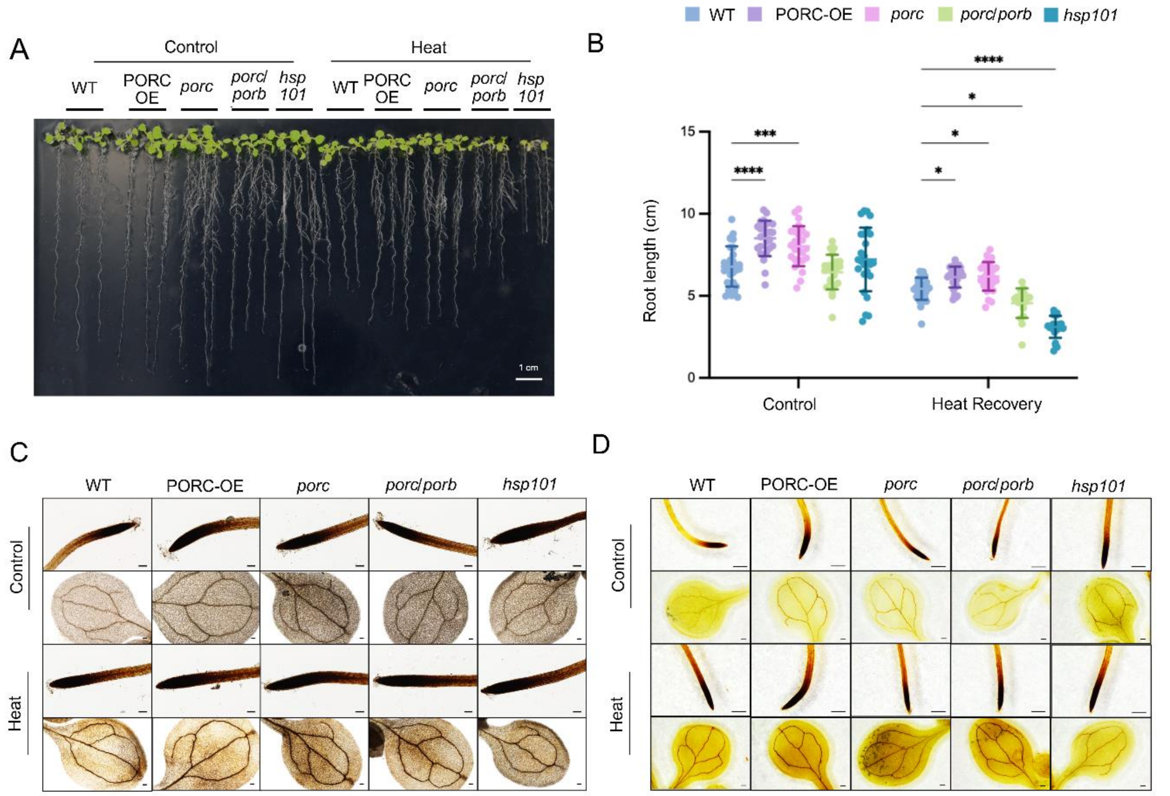

Supplementary Figure 5. Effect of heat shock on seven-day-old seedlings of *porc* transgenic lines. **A.** Phenotype analysis of root development of Arabidopsis wild type, and *porc* transgenic lines after heat shock treatment. **B.** Quantifications of the root length. Graph represents mean values and SD (n=25,*: $p \leq 0.05$, ****: $p < 0.0001$, Two-way ANOVA test). ROS accumulation in Arabidopsis wild type, and *porc* transgenic lines after heat treatment of seven- and ten-day-old seedlings (**C**, **D**) respectively, as revealed by DAB staining. Scale bar = 1mm.

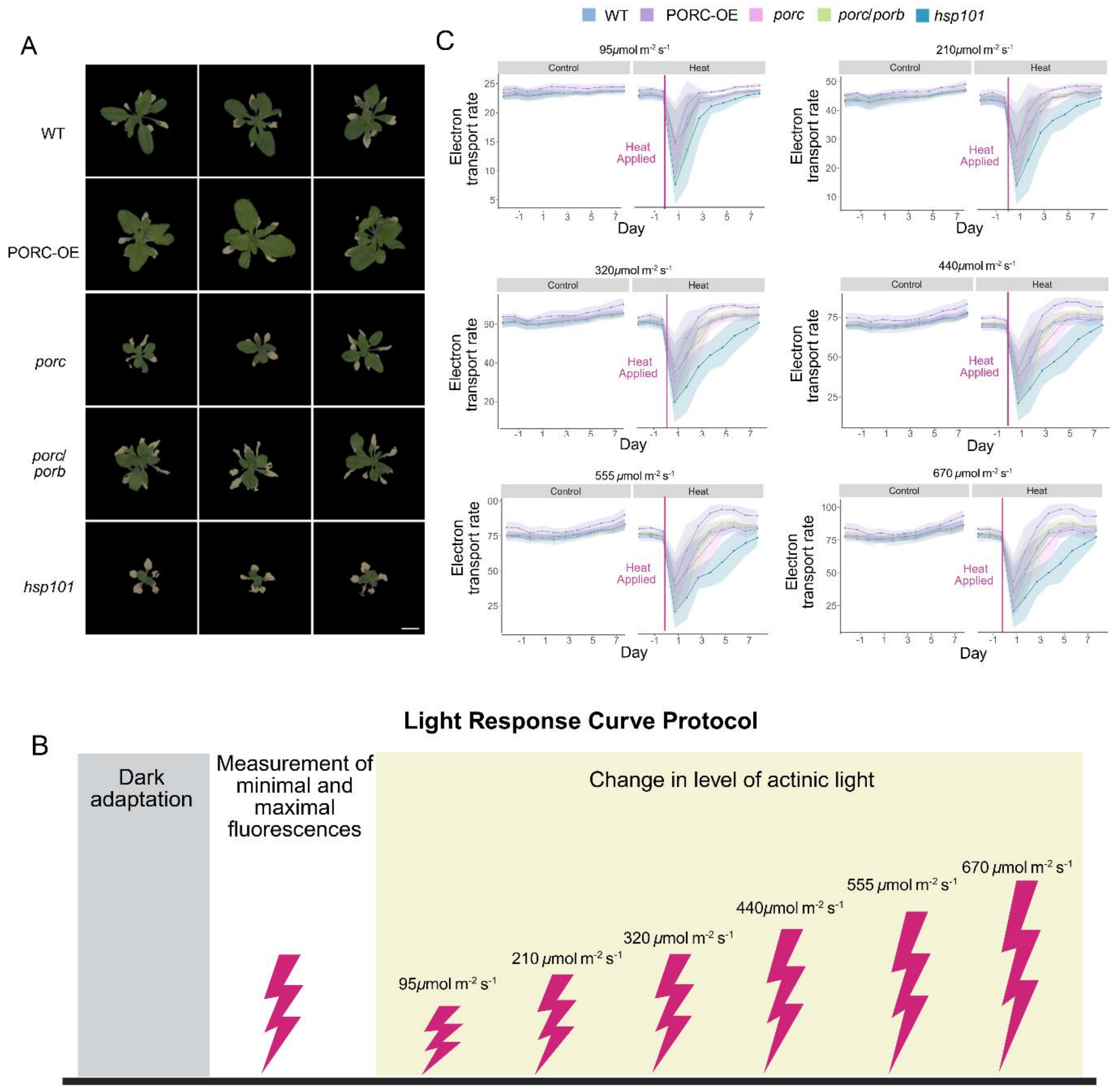

Supplementary Figure 6. Impact of heat shock on photosynthetic activity. A. Representative images of each genotype after the heat recovery. Images were digitally extracted for comparison. Scale bar = 1cm. **B.** Schematic representation of the light response curve protocol. **C.** Comparison of electron transport rates across all tested genotypes using the light response curve protocol (n=18).

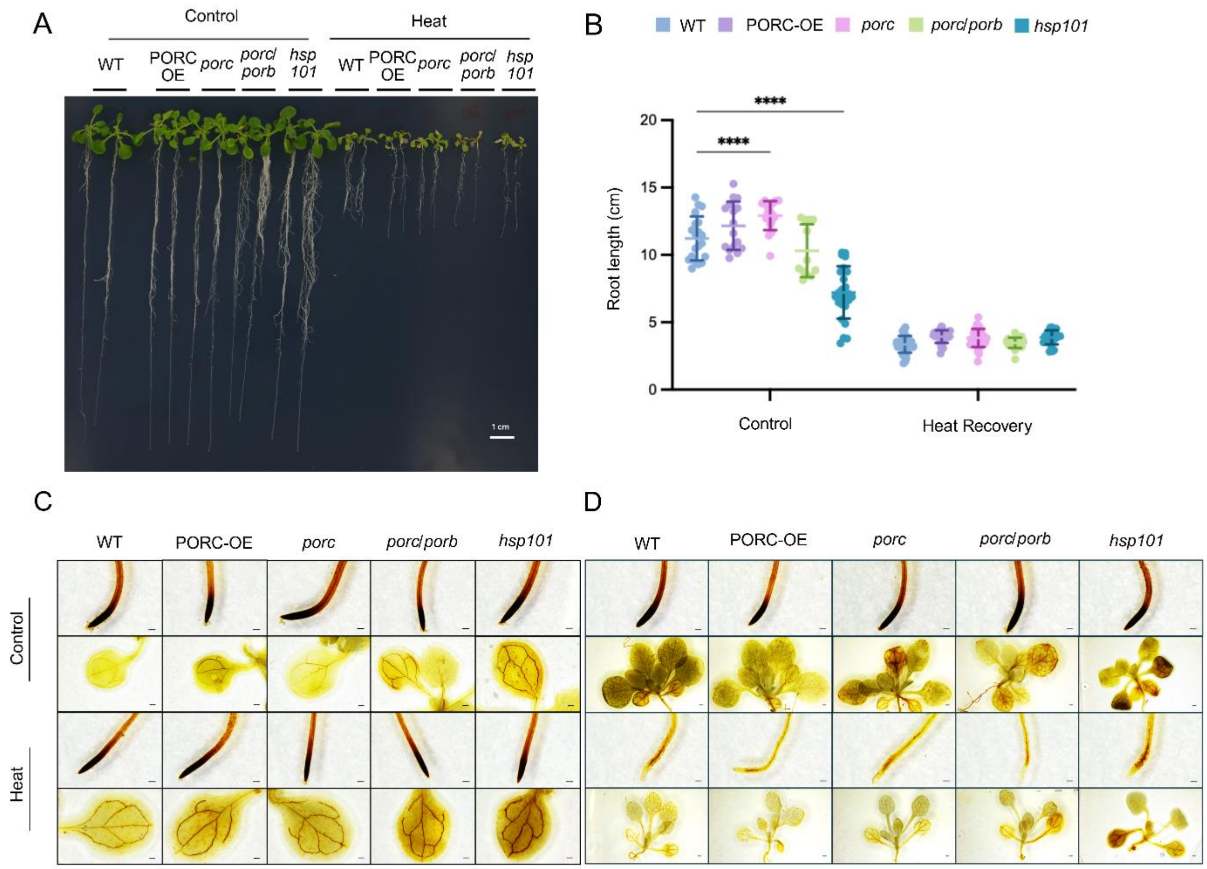

Supplementary Figure 7. Effect of prolonged heat exposure on seven-day-old seedlings of *porc* transgenic lines. A. Phenotype analysis of root development of Arabidopsis wild type, and *porc* transgenic lines after heat shock treatment. **B.** Quantifications of the root length. Graph represents mean values and SD, (n=25,* : $p \leq 0.05$, ****: $p < 0.0001$, Two-way ANOVA test). ROS accumulation in Arabidopsis wild type, and *porc* transgenic lines after five day of prolonged heat treatment on seven- and ten-day-old seedlings (**C, D**) respectively, as revealed by DAB staining. Scale bar = 1mm.

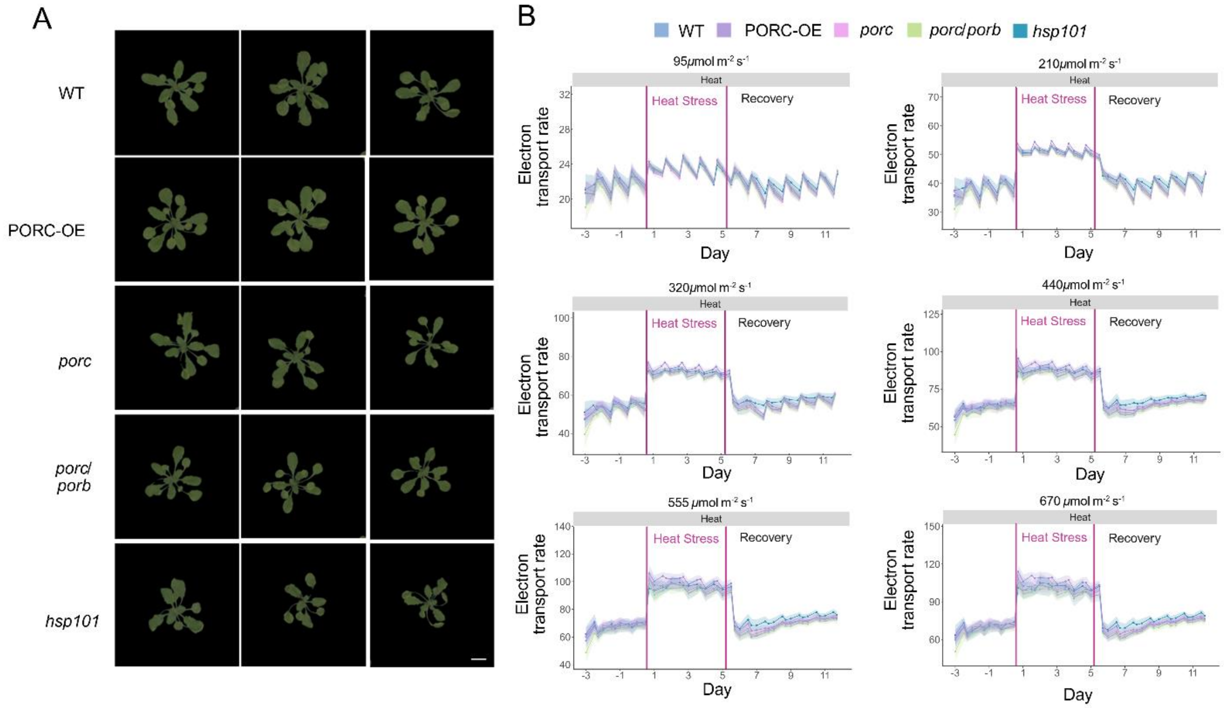

Supplementary Figure 8. Impact of prolonged heat on photosynthetic activity. A. Representative images of each genotype after the heat recovery. Images were digitally extracted for comparison. Scale bar = 1 cm. **B.** Comparison of electron transport rates across all tested genotypes using the light response curve protocol (n=26).

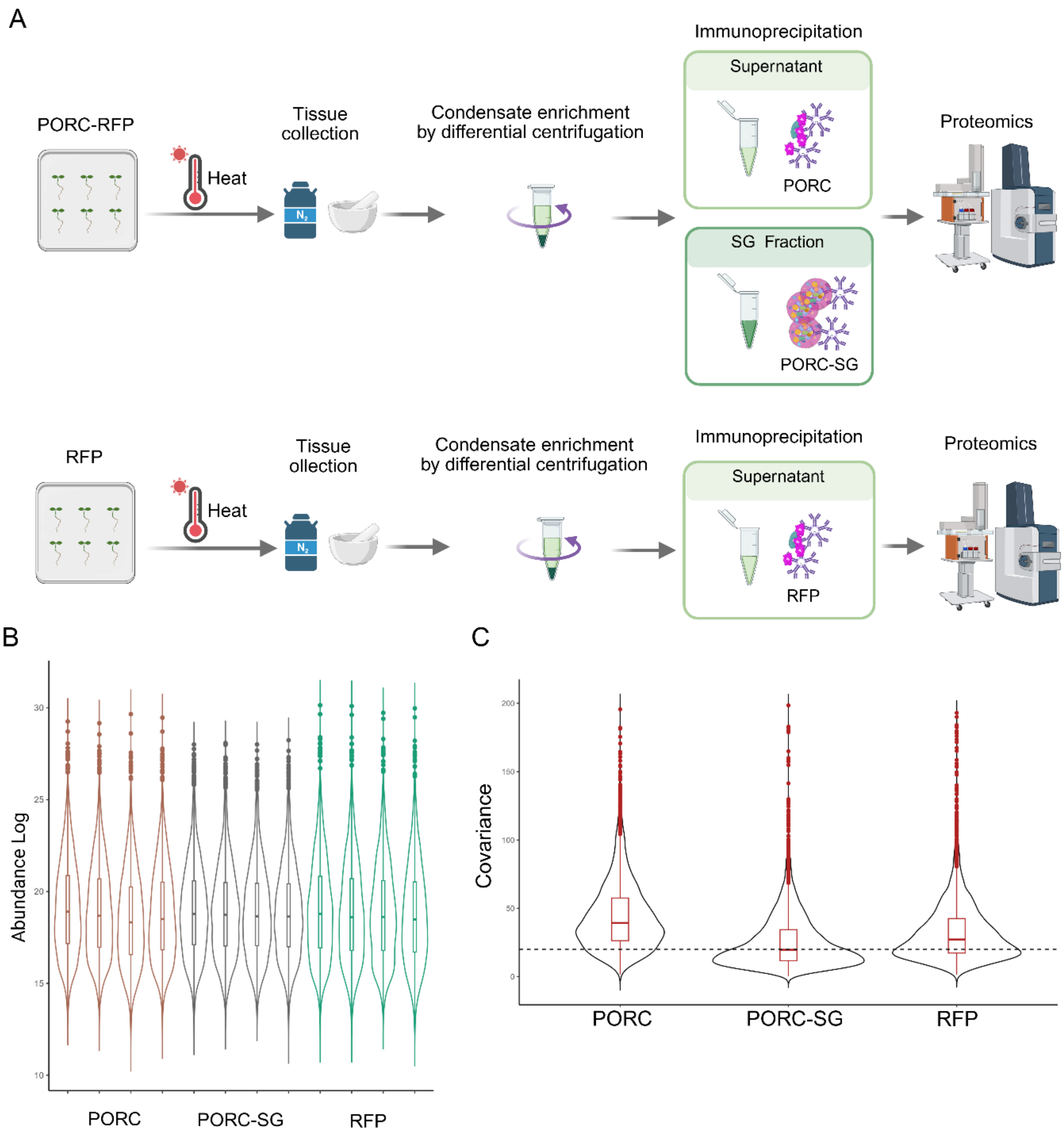

Supplementary Figure 9. Proteomics experimental setup and quality assessment. A. Experimental set up for PORC-SG isolation from POR-RFP line, and the control lines of RFP alone. **B.** Violin plots of log-transformed proteins abundance across samples replicates. Central lines indicate median values (n=4/ condition). **C.** Coefficient of variation (CoV) analysis of tested groups Central lines indicate median values, coefficient of variation for stress granules <20%.
